# Supplementary material for: Releasing Activity Disengages Cohesin’s Smc3/Scc1 Interface in a Process Blocked by Acetylation
Source: Mol Cell. 2016 Feb 18;61(4):563–74. doi: 10.1016/j.molcel.2016.01.026 (PMC4769318; doi:10.1016/j.molcel.2016.01.026)
Supplement: Document S1. Figures S1–S4 and Supplemental Experimental Procedures [file mmc1.pdf]

Molecular Cell, Volume *61*

## Supplemental Information

# Releasing Activity Disengages Cohesin's Smc3/Scc1 Interface in a Process Blocked by Acetylation

**Frederic Beckouët, Madhusudhan Srinivasan, Maurici Brunet Roig, Kok-Lung Chan, Johanna C. Scheinost, Paul Batty, Bin Hu, Naomi Petela, Thomas Gligoris, Alexandra C. Smith, Lana Strmecki, Benjamin D. Rowland, and Kim Nasmyth**

Figure S1

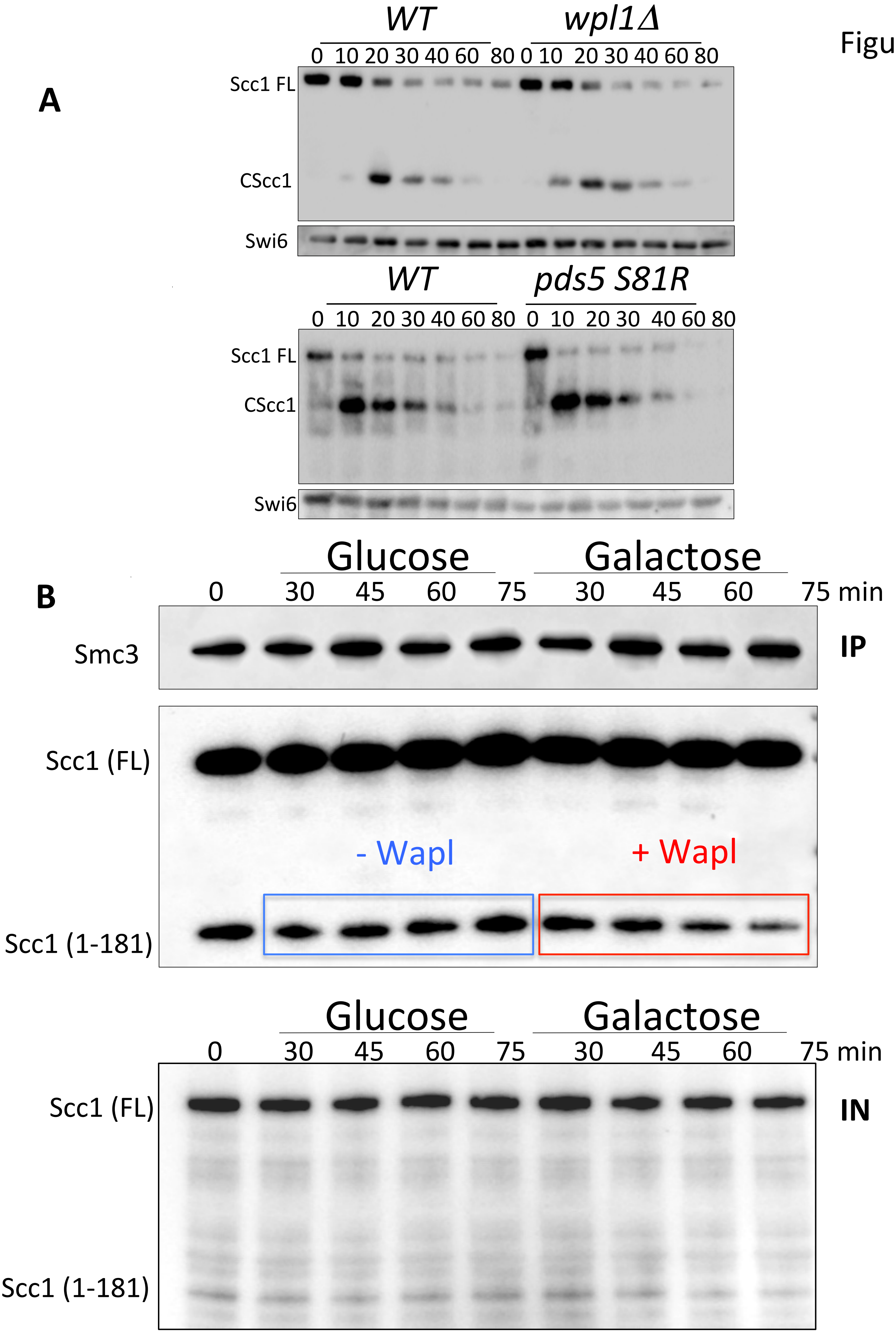
A
WT
wpl1Δ
0 10 20 30 40 60 80 0 10 20 30 40 60 80
Scc1 FL
CScc1
Swi6
WT
pds5 S81R
0 10 20 30 40 60 80 0 10 20 30 40 60 80
Scc1 FL
CScc1
Swi6
B
Glucose
Galactose
0 30 45 60 75 30 45 60 75 min
Smc3
IP
Scc1 (FL)
- Wapl
+ Wapl
Scc1 (1-181)
Glucose
Galactose
0 30 45 60 75 30 45 60 75 min
Scc1 (FL)
IN
Scc1 (1-181)

Figure S2

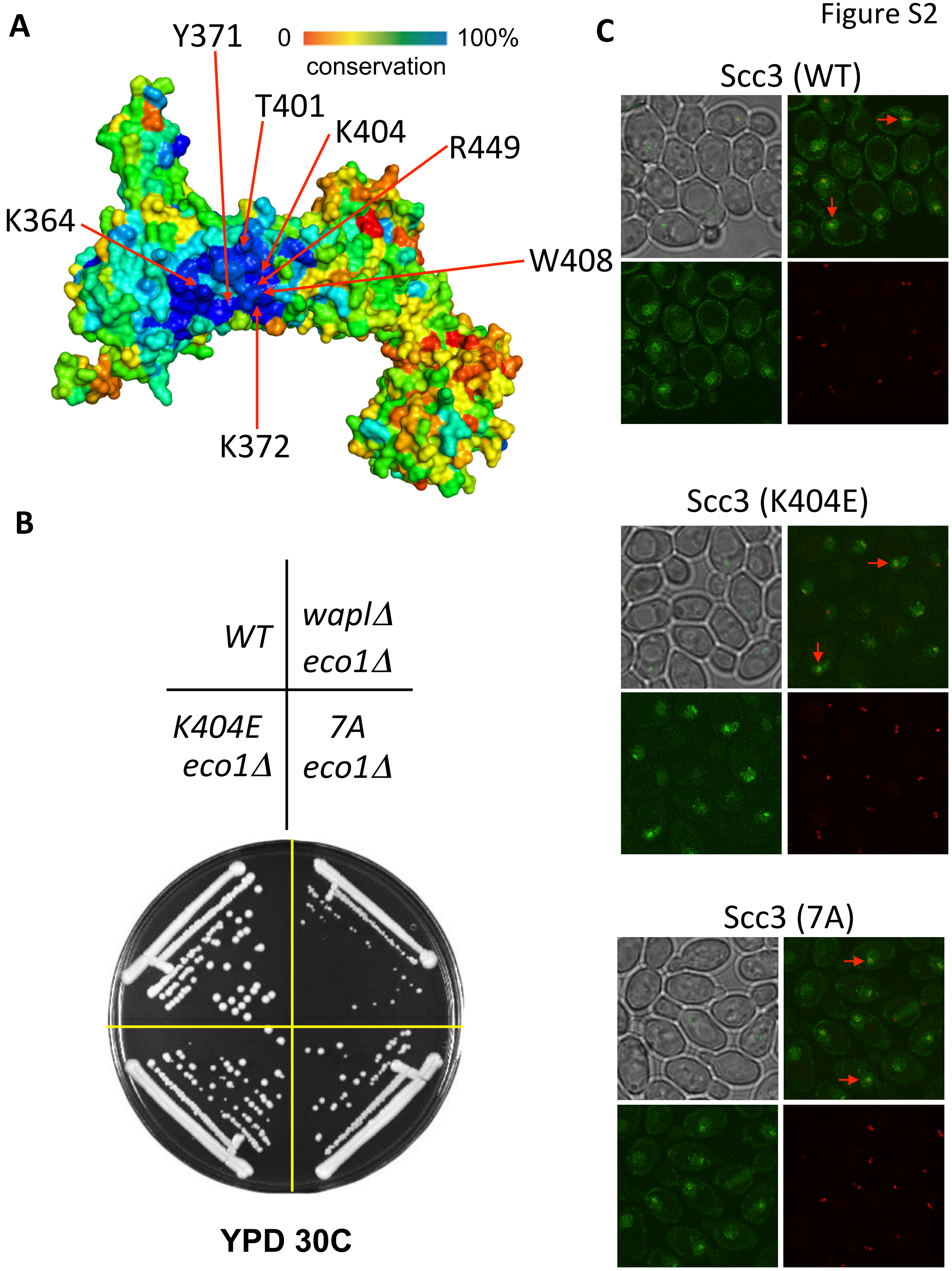

A
Y371
0
100%
conservation
T401
K404
R449
K364
W408
K372
B
WT
waplΔ
eco1Δ
K404E
eco1Δ
7A
eco1Δ
YPD 30C
C
Scc3 (WT)
Scc3 (K404E)
Scc3 (7A)

Figure S3

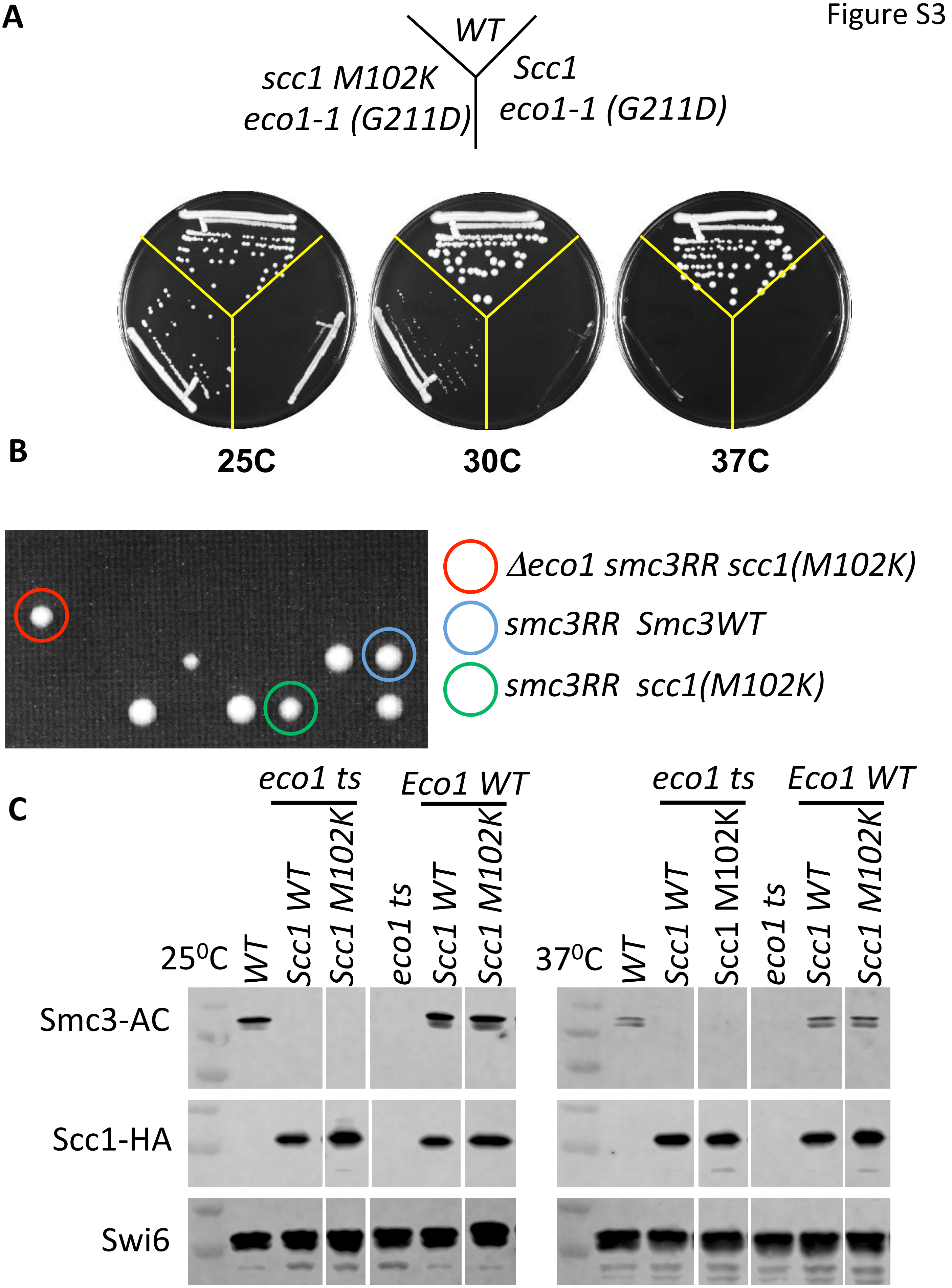
A
WT
scc1 M102K
eco1-1 (G211D)
Scc1
eco1-1 (G211D)
25C
30C
37C
B
Δeco1 smc3RR scc1(M102K)
smc3RR Smc3WT
smc3RR scc1(M102K)
C
eco1 ts
Eco1 WT
eco1 ts
Eco1 WT
25⁰C
WT
Scc1 WT
Scc1 M102K
eco1 ts
Scc1 WT
Scc1 M102K
37⁰C
WT
Scc1 WT
Scc1 M102K
eco1 ts
Scc1 WT
Scc1 M102K
Smc3-AC
Scc1-HA
Swi6

Figure S4

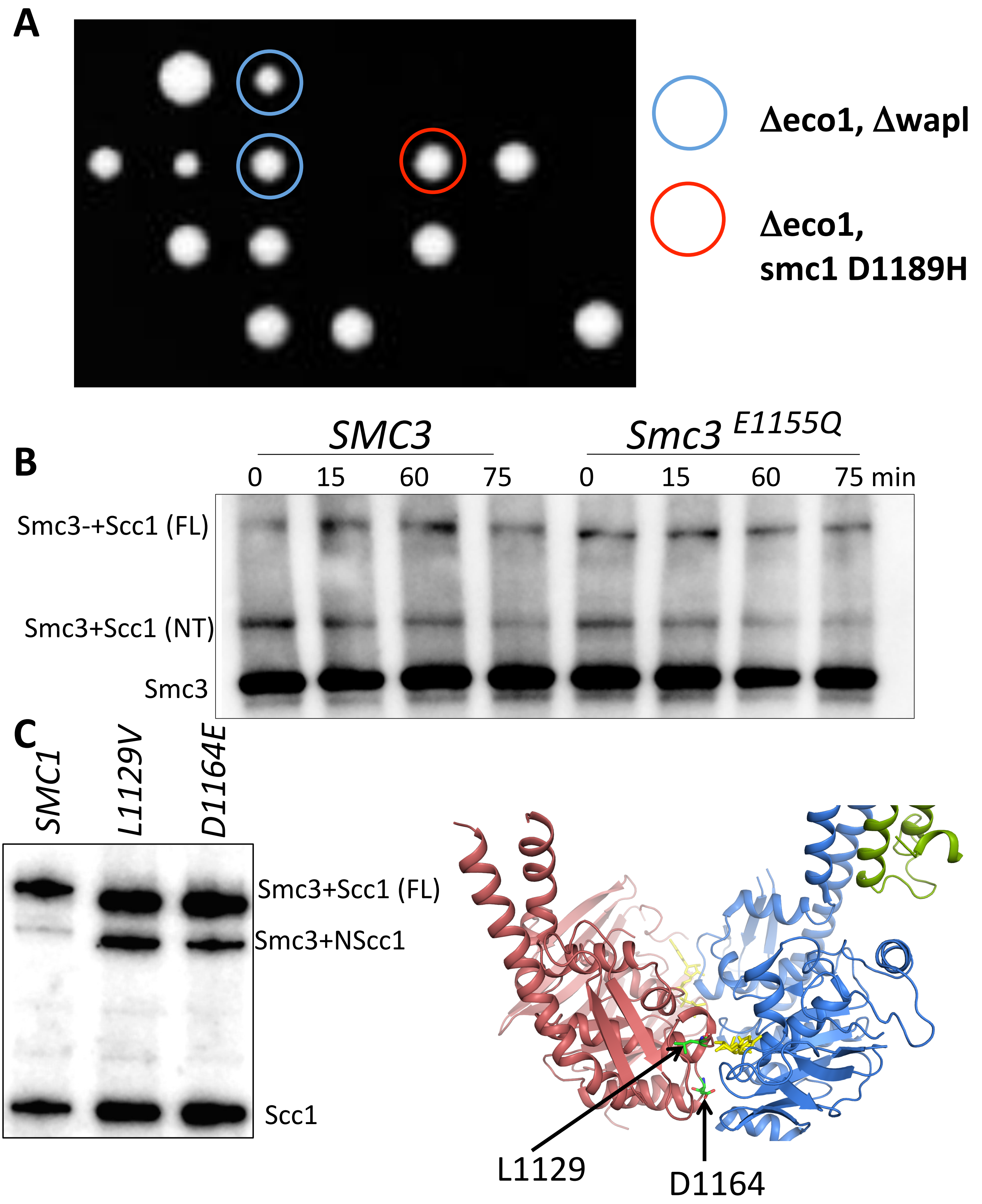
A
Δeco1, Δwapl
Δeco1,
smc1 D1189H
B
SMC3
Smc3 E1155Q
0
15
60
75
0
15
60
75 min
Smc3-+Scc1 (FL)
Smc3+Scc1 (NT)
Smc3
C
SMC1
L1129V
D1164E
Smc3+Scc1 (FL)
Smc3+NScc1
Scc1
L1129
D1164

**Supplementary Figure Legends**

**Figure S1. Related to Figures 1 and 2**

**(A) Stability of Scc1 cleavage fragments in releasing activity mutants.** Wild type (K17960), *wpl1Δ* (K20236) and *pds5-S81R* (K20521) strains expressing *CDC20* from the *GAL* promoter were grown to logarithmic phase at $25^0$C in YP medium containing galactose , transferred to galactose free media to induce metaphase arrest (time 0), and anaphase triggered by galactose re-addition. Separase cleavage of Scc1 was followed by Western blotting, C-terminal HA epitopes.

**(B) Wapl triggers dissociation of NScc1 from Smc3** HOS1 or *hos1Δ* strains containing galactose inducible *WPL1* gene, K22555 (*MATa SMC3 (S1043C)-HA6 MYC3-SCC1(TEV268) pGAL1-10-WPL1*) and K22810 (MAT*a hos1Δ SMC3(S1043C)-HA6 MYC3-SCC1(TEV268) pGAL1-10-WPL1*) were grown in YP Raffinose medium at $25^0$C and were arrested in nocodazole for 2 hours. Galactose was then added to induce Wapl expression. Samples were taken at the indicated time points to immunoprecipitate Smc3 and analysed by Western blotting using anti MYC antibodies. The structure of Smc3-Scc1NTD complex PDB:4UX3 is shown with Scc1 C56, Smc3 S1043 residues marked.

**Figure S2. Related to Figure 4. Scc3's highly conserved surface is essential for release**

**(A)** Surface conservation of Scc3 orthologs projected on the surface of Zr *Scc3* (blue most and red least conserved) highlighting the conserved K364, Y371, K372, T401, K404, W408 and R449 (*S. cerevisiae*) surface residues that were mutated (7A). **(B)** A YPD plate with Strain K699 (W3031a), K16432 (*eco1Δ wpl1Δ*), K23905 (*eco1Δ scc3 K404E*) and K23908 (*eco1Δ scc3 7A*) grown at $30^0$C shows the suppression of *eco1Δ* by *scc3* (*K404E* and *7A*) mutants. **(C)** Shows live cell imaging of strains K23851 (*WPL1-eGFP MTW1-RFP*), K23854 (*WPL1-eGFP MTW1-RFP scc3 K404E*) and K23852 (*WPL1-eGFP MTW1-RFP scc3 7A*) that were grown to exponential phase in YPD at $25^0$C . GFP (Wapl) RFP (Mtw1) and DIC channels are shown.

**Figure S3. Related Figure 5. Role of residues within Scc1's NTD in the release mechanism**

**(A)** YPD plates with Strain K699 (W3031a), K24507 (*SCC1-HA eco1−1(G211D)*), and K24514 (*SCC1 M102K-HA eco1−1( G211D )*) were grown at $25^0$C, $30^0$C and $37^0$C. **(B)** Diploid (*MATa/α SMC3 smc3Δ TRP1::smc3 K112R K113R Eco1 eco1Δ scc1Δ SCC1 M102K*) was sporulated, tetrads dissected, and selected haploid segregants with their genotypes shown. **(C)** Exponentially growing strains K699 (W3031a), K20307 (*SCC1-HA eco1-1*), K20311 (*SCC1M102K-HA eco1-1*), K16300 (*eco1-1*), K18879 (*SCC1-HA*) and K18881 (*SCC1M102K-HA*), grown at either permissive $25^0$C or the non permissive $37^0$C were lysed and the levels of Smc3 acetylation (Smc3-AC) was detected using antibody that recognises Smc3 K112 K113 AC. Scc1 levels was measured using anti HA antibody and Swi6 was used as loading control. The data are from the same western blot with irrelevant lanes removed.

**Figure S4. Related to Figure 5 and 7**

**(A) Role of residues within Smc3's ATPase domain in release**

Diploids (*MATa/α scc1Δ LEU2::MYC-SCC1 TRP1::smc3 K112R K113R D1189H eco1Δ wpl1Δ*) was sporulated, tetrads dissected, and selected haploid segregants with their genotypes shown.

**(B) Disengagement of NScc1 from Smc3 requires a single round of ATP hydrolysis**

Strains K23567 (*MATa SMC3 URA3::SMC3 S1043C-HA6 MYC3-SCC1(TEV268) pGAL1-10-WPL1*) and K23566 (*MATa SMC3 URA3::smc3(S1043C E1155Q-HA6 MYC3-SCC1(TEV268), pGAL1-10-WAPL*) were grown in YP Raffinose medium at $25^0$C and were arrested in nocodazole for 2 hours. Galactose was then added to induce Wapl expression. Samples were taken at the indicated time points to induce *in vivo* cross-linking with 5mM BMOE. Cross-linking was analysed by Western blotting using anti HA antibodies.

**(C) Disengagement of NScc1 from Smc3 involves engagement of Smc ATPase heads**

Exponentially growing strains K24297 (*MATa SMC3 S1043C::ADE2 MYC3-SCC1(TEV268) SMC1)*, K24523 (*MATa SMC3 S1043C::ADE2 MYC3-SCC1(TEV268) smc1 L1129V)* and K24521 (*MATa SMC3 S1043C::ADE2 MYC3-*

*SCC1(TEV268) smc1 D1164E)* were treated as described in Figure 6(C) and analysed by western blotting using anti MYC antibodies. The positions of the Smc1 L1129 and D1164 residues are marked in a model of Smc1 and Smc3 head domains that are in an engaged state.

**Strain construction**

The 7A mutant version of scc3 (under its native promoter) was incorporated at the leu2 locus in heterozygous SCC3/scc3Δ diploids cells. Diploids were sporulated and tetrads dissected at 23 °C on YPD plates. The genotype of the resulting haploids was determined by replica plating, and viable cells with only the ectopic copy were used in this manuscript. The single mutants K404E, K404A, Y405E and Y405A on scc3 were generated modifying the genome in *S.cerevisiae* using the CRISPR-Cas9 systems. For this we co-transformed the cells with a 600nt PCR "healing fragment" along with a 2 micron LEU2 plasmid constitutively expressing Cas9 endonuclease and transcribing the structural RNA that contains the Cas9 binding site and the guide RNA that will target the double strand break formation by Cas9 to the scc3 gene. The guide RNA was designed 20nt upstream of the NGG (L455, E456) using the annealed oligos MB530F/MB530R (MB530F: 5'-ATCACGCTTCAAAACTAAGATCC-3' , MB531R: 5'- AACGGATCTTAGTTTTGAAGCGT-3') and fused afterwards at the 5' end of the structural RNA with a Sap1 cloning. The 600nt PCR "healing fragment" containing the single mutants, a mutagenized NGG Cas9 sequence target (L455, E456: C**TGG**AA to C**TAG**AA) and 5'-, 3'- homology arms by which to repair the double strand break created by Cas9 was created using the oligos MB528F/MB529R (MB528F: 5-CTTGTGTTGGATTTGTTGAC-3', MB529R: 5'-TGGATACTGTGGATCCTTACATCAAGATTAACGTCACGGATTGCCACTTCT AGGATCTTAGTTTTGAAGCG-3' ). As a control, no colonies were obtained after yeast transformation of the 2 micron Leu2 plasmid alone without the "healing fragment". All mutations were confirmed afterwards by genome sequencing.

**In vivo chemical cross-linking**

Strains were grown in YEPD at 25 °C to $OD_{600nm}$ = 0.5-0.6 and arrested in nocodazole for 1.5 h. 30 OD units were washed in ice-cold PBS, then re-suspended in 1.5 ml ice-cold PBS. The suspensions were then split into 2 x 750 μl and 35 μl BMOE (stock: 125 mM in DMSO, 5 mM final) or DMSO was added for 6 min on ice. Cells were washed with 2 x 2 ml ice-cold PBS containing 5 mM DTT, resuspended in 500 μl EBX lysis buffer containing 1 mM DTT, benzonase (1:1000), RNase (0.1 mg/ml), Roche Complete Protease Inhibitors (2X) and PMSF (1 mM), lysed in a FastPrep-24 (MP Biomedicals) for 3 min at 6 m/s with 500 μl of acid-washed glass beads (425-

600 μm, Sigma) and lysates cleared (5 min, 12 k*g*). Protein concentrations were adjusted after Bradford assay and cohesin immuno-precipitated using anti-PK antibody (AbD Serotec, 1 h, 4 °C), anti MYC antibody (Santa cruz1 h, 4 °C), and protein G dynabeads (1 h, 4 °C with rotation) or Anti HA affinity resin (Roche, 1 h, 4 °C). Beads were washed with 2 x 1 ml lysis buffer, resuspended in 50 μl 2x sample buffer, incubated at 65°C for 5 min and the supernatant loaded onto a either 3-8% or 4-12% gradient gels (life technologies).

**Mini Chromosome IPs**

Strains containing a 2.3KB minichromosome were grown in YEPD at 25 °C and processed for in vivo crosslinking like mentioned above. For Southern blotting, pull-downs of lysates (in 25 mM Hepes pH 8.0, 50 mM KCl, 50 mM $MgSO_4$, 10 mM trisodium citrate, 25 mM sodium sulfite, 0.25% triton-X, Roche Protease Inhibitor, 1 mM PMSF, 2 mM DTT, 0.1 mg/ml RNase) containing a circular 2.3 kb mini-chromosome were performed as above, DNA was eluted in 30 μl 1% SDS with DNA loading dye (65 °C, 4 min) and run on 0.8% agarose gels containing ethidium bromide (1.4 V/cm, 22h, 4 °C).

**Protein Purification**

*E. coli* BL21(DE3) RIPL cells (Stratagene) were transformed with pET vectors containing the *S. cerevisiae* Scc3 WT, Scc3 E404K and Wapl genes and induced at $OD_{600}$ 0.6 with 1 mM IPTG at 23 °C over night. The cells were pelleted and re-suspended with TAP buffer (50 mM Tris–HCl, 250 mM NaCl, 1 mM β-mercaptoethanol, pH 7.5) adding EDTA-free protease inhibitor cocktail tablets (Roche). After re-suspension, and one round of cell lysis at 18 KPsi (Constant Systems), 1 min sonication at 80% AMPL (Sonics Vibra-Cell) were performed. Cell lysate was pre-cleared by centrifugation at 4000 rpm for 15 min, and supernatant was cleared by centrifugation at 15,000 rpm for 1 h at 4 °C (Beckman Coulter, rotor JLA-16.250). All purified proteins are $His_6$ tagged and supernatants were incubated with Talon Superflow beads (Clontech) for 3 h at 4 °C. Beads were washed 3 times with TAP buffer containing 10 mM imidazole. Proteins were eluted in TAP buffer with 500 mM imidazole and loaded onto a Superdex 200 16/60 chromatography column

(GE Healthcare) equilibrated with TAP buffer (50 mM Tris–HCl, 100 mM NaCl, 1 mM β-mercaptoethanol, pH 7.5). Peak fractions were collected and concentrated using Vivaspin columns (Sartorius Stedim Biotech).

**Gel filtration for Protein Binding**

For in vitro binding assays, 15 μM of each pure protein were incubated at 4 °C for 2–3 h, at a final volume of 150 μl in TAP buffer (50 mM Tris–HCl, 100 mM NaCl, 1 mM β-mercaptoethanol, pH 7.5) and then loaded onto a Superose 6 (GE Healthcare) column. 15 μl protein fractions were collected and analyzed by 3-8% SDS–PAGE and Coomassie staining.
