# Supplementary material for: Releasing Activity Disengages Cohesin’s Smc3/Scc1 Interface in a Process Blocked by Acetylation
Source: Mol Cell. 2016 Feb 18;61(4):563–74. doi: 10.1016/j.molcel.2016.01.026 (PMC4769318; doi:10.1016/j.molcel.2016.01.026)
Supplement: Document S2. Article plus Supplemental Information [file mmc2.pdf]

Molecular Cell

Article

Releasing Activity Disengages Cohesin's Smc3/Scc1 Interface in a Process Blocked by Acetylation

Graphical Abstract

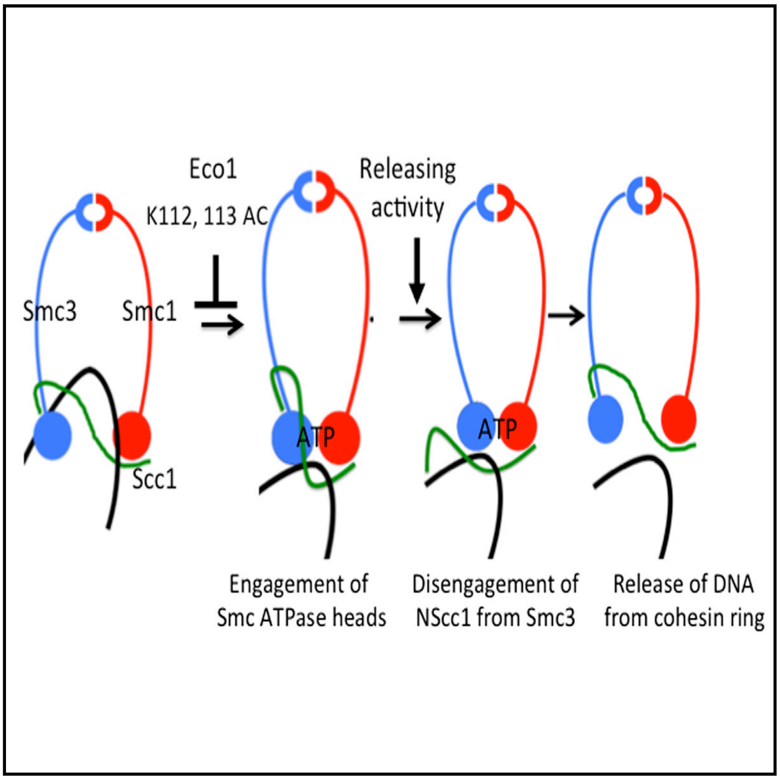

Authors

Frederic Beckouët,
Madhusudhan Srinivasan,
Maurici Brunet Roig, ..., Lana Strmecki,
Benjamin D. Rowland, Kim Nasmyth

Correspondence

kim.nasmyth@bioch.ox.ac.uk

In Brief

Cohesin entraps sister DNAs within a ring formed by its Scc1, Smc1, and Smc3 subunits. Beckouët et el. have shown that cohesin's release from the DNA is mediated by disengagement of the Smc3/Scc1 interface. This process involves interaction between the SMC ATPase heads and is inhibited by Smc3 acetylation.

Highlights

- Releasing activity disengages cohesin's Smc3/Scc1 interface
- Releasing activity requires an interaction between the SMC ATPase domains
- Smc3 acetylation inhibits releasing activity

Accession Numbers

GSE76890

Beckouët et al., 2016, Molecular Cell *61*, 563–574
February 18, 2016 ©2016 The Authors
http://dx.doi.org/10.1016/j.molcel.2016.01.026

Releasing Activity Disengages Cohesin's Smc3/Scc1 Interface in a Process Blocked by Acetylation

Frederic Beckouët,[1,5,6] Madhusudhan Srinivasan,[1,6] Maurici Brunet Roig,[1,6] Kok-Lung Chan,[2,6] Johanna C. Scheinost,[1] Paul Batty,[1] Bin Hu,[3] Naomi Petela,[1] Thomas Gligoris,[1] Alexandra C. Smith,[1] Lana Strmecki,[1] Benjamin D. Rowland,[4] and Kim Nasmyth[1,*]
[1]Department of Biochemistry, University of Oxford, South Parks Road, Oxford OX1 3QU, UK
[2]Genome Centre, University of Sussex, Sussex House, Brighton BN1 9RH, UK
[3]Department of Molecular Biology and Biotechnology, University of Sheffield, Firth Court, Western Bank, Sheffield S10 2TN, UK
[4]Division of Cell Biology, The Netherlands Cancer Institute, Plesmanlaan 121, 1066 CX Amsterdam, the Netherlands
[5]Laboratoire de Biologie Moléculaire Eucaryote, UMR 5099 University Paul Sabatier Toulouse III CNRS, 118, Route de Narbonne, 31062 Toulouse, France
[6]Co-first author
*Correspondence: kim.nasmyth@bioch.ox.ac.uk
http://dx.doi.org/10.1016/j.molcel.2016.01.026
This is an open access article under the CC BY license (http://creativecommons.org/licenses/by/4.0/).

SUMMARY

Sister chromatid cohesion conferred by entrapment of sister DNAs within a tripartite ring formed between cohesin's Scc1, Smc1, and Smc3 subunits is created during S and destroyed at anaphase through Scc1 cleavage by separase. Cohesin's association with chromosomes is controlled by opposing activities: loading by Scc2/4 complex and release by a separase-independent releasing activity as well as by cleavage. Coentrapment of sister DNAs at replication is accompanied by acetylation of Smc3 by Eco1, which blocks releasing activity and ensures that sisters remain connected. Because fusion of Smc3 to Scc1 prevents release and bypasses the requirement for Eco1, we suggested that release is mediated by disengagement of the Smc3/Scc1 interface. We show that mutations capable of bypassing Eco1 in Smc1, Smc3, Scc1, Wapl, Pds5, and Scc3 subunits reduce dissociation of N-terminal cleavage fragments of Scc1 (NScc1) from Smc3. This process involves interaction between Smc ATPase heads and is inhibited by Smc3 acetylation.

INTRODUCTION

Sister chromatid cohesion essential for chromosome segregation is mediated by a multisubunit complex called cohesin (Guacci et al., 1997; Michaelis et al., 1997), which contains two SMC proteins, Smc1 and Smc3, and an α-kleisin subunit Scc1. Both Smc proteins form 50-nm-long intramolecular antiparallel coiled coils with a hinge/dimerization domain at one end and at the other an ATPase head domain formed from the protein's N- and C-terminal sequences. They bind each other via their "hinges" to form V-shaped Smc1/Smc3 heterodimers (Nasmyth and Haering, 2005). Most remarkable is the manner by which the α-kleisin subunit binds to the ATPases at the vertices of this heterodimer. A pair of α helices within Scc1's N-terminal domain (NTD) forms a four-helical bundle with the coiled coil emerging from Smc3's ATPase head (Gligoris et al., 2014), while a winged helix within its C-terminal domain (CTD) binds the base of Smc1's ATPase, thereby creating a huge asymmetric tripartite ring. Sister chromatid cohesion is thought to be mediated by entrapment of sister DNAs within these rings (Haering et al., 2002), a concept known as the ring model. Bacterial Smc/kleisin complexes also form very similar tripartite rings (Bürmann et al., 2013) that entrap DNAs (Wilhelm et al., 2015), raising the possibility that all Smc/kleisin complexes operate as topological devices.

Coentrapment of sister DNAs within cohesin rings (Gligoris et al., 2014; Haering et al., 2008) takes place during replication and is accompanied by acetylation of a pair of conserved lysine residues within Smc3's ATPase domain (K112 and K113) by an acetyltransferase called Eco1 (Ivanov et al., 2002; Nasmyth and Haering, 2009). Smc3 acetylation is essential for establishment of stable cohesion. It is maintained throughout G2 and M phases and only removed by a class I deacetylase called Hos1 in yeast and HDAC8 in mammalian cells (Beckouët et al., 2010; Rolef Ben-Shahar et al., 2008; Deardorff et al., 2012) upon cleavage of Scc1 by separase at anaphase onset, an event that opens the ring and destroys the connection between sister DNAs, triggering sister chromatid disjunction (Uhlmann et al., 1999).

Cohesin's association with DNA, known as cohesin loading, depends on the ability of the ring to hydrolyse ATP bound to Smc1 and Smc3 (Arumugam et al., 2003, 2006), a process facilitated by the activity of a separate complex called Kollerin, which contains the Scc2 and Scc4 proteins (Ciosk et al., 2000). According to the ring model, loading involves passage of DNAs into the ring, which is proposed to take place via a gate created by transient dissociation of the Smc1/Smc3 hinge interface (Gruber et al., 2006). Cohesin rings can entrap in this manner either single DNA molecules or, following replication, a pair of sister DNAs (Gligoris et al., 2014).

Two mechanisms account for cohesin's release from chromosomes. Best understood is cleavage of its kleisin subunit by separase (Uhlmann et al., 2000). The N- and C-terminal Scc1

fragments associated with Smc3 and Smc1 ATPase heads, respectively (Gruber et al., 2003), are subsequently degraded as daughter cells enter a new cell cycle. Degradation of the C-terminal fragment is mediated by the Ubr1 ubiquitin protein ligase (Rao et al., 2001), but the mechanism responsible for destroying the N-terminal fragment has yet to be elucidated. The second mechanism is independent of separase but requires a regulatory subunit associated with cohesin called Wapl (Gandhi et al., 2006; Kueng et al., 2006). It was initially called the prophase pathway because the process is greatly accelerated in animal cells as they enter mitosis and accounts for the release of most cohesin from chromosome arms during this stage of the cell cycle.

It turns out that a releasing mechanism related to the prophase pathway operates throughout the cell cycle and is responsible for cohesin's turnover on interphase chromatin, not only in animal cells (Gerlich et al., 2006) but also in yeast (Chan et al., 2012), where prophase-specific release does not occur and the entire pool of chromosomal cohesin is cleaved by separase during anaphase. In addition to Wapl, releasing activity depends on K112 and K113 within Smc3 (in their unmodified state) and on two large regulatory subunits called Pds5 and Scc3, which bind, respectively, to sequences within the N- and C-terminal halves of Scc1 (Chan et al., 2013; Hara et al., 2014; Roig et al., 2014; Rowland et al., 2009).

Because it has the potential to destroy sister chromatid cohesion, releasing activity must somehow be neutralized after replication, at least for the chromosomal cohesin pool destined to hold sisters stably together during mitosis, and this is the function of acetylation of K112 and K113 by Eco1. In yeast, this modification appears sufficient to block releasing activity, but in animal cells it requires in addition recruitment of sororin (Nishiyama et al., 2010; Rankin et al., 2005). Key to the concept that the function of acetylation is to neutralize releasing activity was the finding that mutations within Wapl, Smc3, Pds5, and Scc3 that bypass the lethality of *eco1* mutants (Rowland et al., 2009; Tanaka et al., 2001) are all defective in releasing activity (Chan et al., 2012). Likewise, inactivation of Wapl bypasses the need for sororin in animal cells or Eco1 orthologs in animals (Nishiyama et al., 2010) and plants (De et al., 2014).

If loading is synonymous with entrapment of DNAs within cohesin rings, then release must involve their subsequent escape. The cohesin ring must have an exit as well as an entry gate for DNAs. A clue to the exit gate's identity was the finding that co-translational fusion of Smc3's C terminus to Scc1's N terminus creates a functional fusion protein that fails to turnover on chromosomes and, like releasing activity mutations, supresses *eco1Δ* lethality (Chan et al., 2012). If the exit gate opened by releasing activity were created by transient disengagement of Scc1's NTD from Smc3's coiled coil, then fusion of the two proteins would create a topological barrier to DNA escape.

Though the properties of cohesin rings containing Smc3-Scc1 fusion proteins are clearly consistent with releasing activity working by disengaging Scc1 from Smc3, it does not address the key issue of whether or not releasing activity actually opens this interface in living cells. Under normal circumstances, this may be difficult to measure, as disengagement is presumably a fleeting process that is swiftly followed by re-engagement of Scc1, whose CTD remains attached to Smc1. However, if releasing activity also operated on the N-terminal cleavage fragments (NScc1) created by separase, disengagement might lead to a permanent and thereby more readily measurable dissociation. We show here that this is indeed the case, namely that releasing activity is responsible for removing NScc1 from Smc3's coiled coil, that this process likely involves engagement of Smc1 and Smc3 ATPases, and that the process is blocked by acetylation of Smc3's K112 and K113 residues. Our findings constitute direct evidence that separase-independent release involves creation of a gate at the Smc3-Scc1 interface from which previously entrapped DNAs can escape.

RESULTS

NScc1 Is Stabilized by Releasing Activity Mutations

We previously demonstrated that Smc3 is deacetylated by Hos1 in response to Scc1 cleavage at the onset of anaphase (Beckouët et al., 2010). If releasing activity disengages Scc1 from unacetylated Smc3, and if it also does so after Scc1 has been cleaved, then the activity should detach irreversibly NScc1 from deacetylated Smc3, and this might stimulate proteolysis. To address this, wild-type and *wpl1Δ* cells arrested in metaphase by Cdc20 depletion were triggered to undergo anaphase and enter G1 (by reinduction of Cdc20). Western blot analysis revealed that *wpl1Δ* greatly delayed degradation of N- (Figure 1A) but not C-terminal fragments (see Figure S1A available online). This effect is not due to loss of Wapl per se, because it was also observed in mutants known to be defective in releasing activity, namely *pds5 S81R* and *scc3 E202K* (Rowland et al., 2009) (Figures 1B and 1C). Note that Wapl is still recruited to chromosomal cohesin complexes containing Scc3 E202K protein, and yet the mutation reduces NScc1 degradation as much as *wpl1Δ*.

Releasing Activity Promotes Dissociation of NScc1 from Smc3

Releasing activity might promote NScc1 degradation either directly, by interacting for example with the relevant proteolytic machinery, or indirectly, by promoting the fragment's release from Smc3, which is a precondition for degradation. To measure the effect of releasing activity on disengagement of NScc1 from Smc3 per se, it is necessary to uncouple disengagement from proteolysis. This might be possible if proteolysis were a cell-cycle-dependent event and only occurred as cells undergo anaphase and enter G1. We therefore compared NScc1's fate in wild-type and *wpl1Δ* cells when generated by induction of tobacco etch virus (TEV) protease in G2/M phase-arrested cells whose Scc1 contained a TEV instead of a separase cleavage site at position 268 (Figure 1D). Interestingly, little or no degradation occurred in either wild-type or *wpl1Δ* mutant cells, at least during a 90 min window following cleavage (Figure 2A). NScc1 proteolysis is either an event associated only with separase cleavage or more likely one that only occurs from anaphase to G1.

Having discovered a condition in which NScc1 is stable, we were in a position to test whether Wapl influences dissociation. To do this, we used a version of Smc3 with a functional cysteine substitution within its coiled coil (S1043C) that can be efficiently crosslinked to a natural cysteine within Scc1's NTD (C56) (Figure 1D) in living cells using the homobifunctional sulfhydryl active reagent Bis-maleimidoethane (BMOE) (Gligoris et al., 2014). In cells arrested in G2/M by nocodazole, the presence or absence

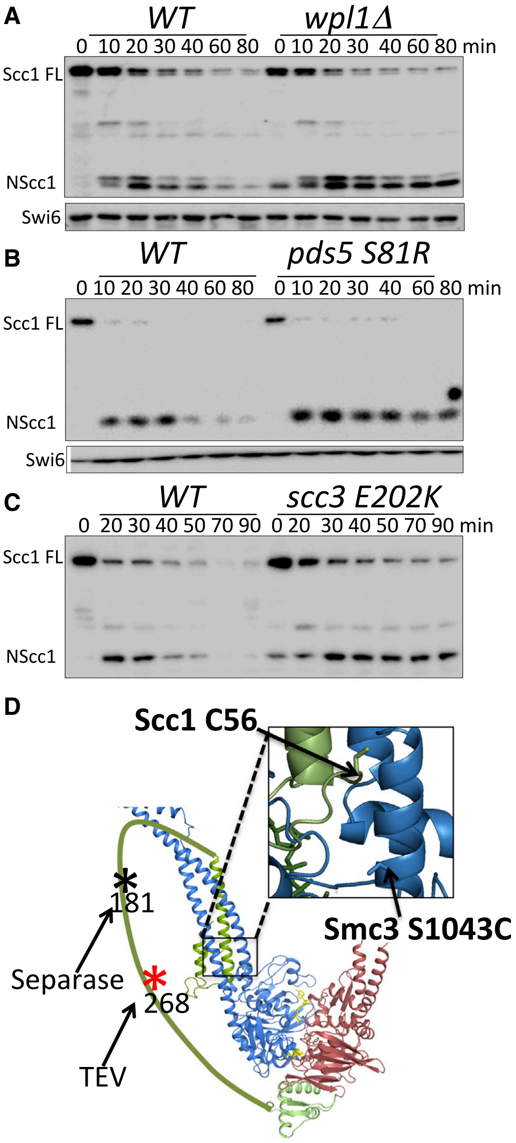

Figure 1. Stability of Scc1 Cleavage Fragments in Releasing Activity Mutants

(A–C) Wild-type (K17960), *wpl1Δ* (K20236), *pds5-S81R* (K20521), and *Scc3-E202K* (K20526) strains expressing *CDC20* from the *GAL* promoter were grown to logarithmic phase at 25°C in YP medium containing galactose, transferred to galactose-free media to induce metaphase arrest (time 0), and anaphase triggered by galactose readdition. Separase cleavage of Scc1 was followed by western blotting, detecting N-terminal Myc tag on Scc1.

(D) Model of the ATPase domains of Smc1 and Smc3 in an engaged state driven by ATP binding. The separase cleavage site in Scc1 at position 181 is marked with a black asterisk; TEV sites at position 268 are marked with a red asterisk.

of Wapl had little or no effect on BMOE-induced crosslinking between full-length (FL) Scc1 C56 and Smc3 S1043C proteins (Figure 2B). The lack of effect is unsurprising. In such cells, a large fraction of Smc3 is acetylated and should not, therefore, be subject to releasing activity. Moreover, it is doubtful that our crosslinking assay would detect transient disengagement of FL Scc1 even within unacetylated complexes.

In *wpl1Δ* mutant but not wild-type cells, 1–181 NScc1 (created by separase; Figure 1D) accumulates to high levels (Figure 2A). Moreover, it is crosslinked to Smc3 with an efficiency similar to FL Scc1 (Figure 2B), implying that NScc1 remains bound to Smc3 long after its creation at the previous anaphase. Induction of TEV protease in G2/M phase cells triggered cleavage of FL protein, creating similar amounts of 1–268 NScc1 (Scc1 TEV) in wild-type and mutant cells (Figure 2A). Despite this equality, treatment with BMOE induced more efficient crosslinking between Smc3 S1043C and C56 within 1–268 NScc1 in *wpl1Δ* than in wild-type (Figure 2B), implying that Wapl promotes dissociation. To exclude the possibility that this is due to a conformational change in the interaction between Scc1 and Smc3 rather than disengagement per se, we measured the amount of 1–268 NScc1 in immunoprecipitates of Smc3 from cells untreated with BMOE (Figure 2C). This confirmed that greater amounts of NScc1 remained associated with Smc3 in *wpl1Δ* than in wild-type cells (Figure 2C). Taken together, these data imply that releasing activity is required to disengage NScc1 from Smc3's coiled coil. Note that because cleavage either by TEV or separase induces Smc3 de-acetylation, NScc1's disengagement from Smc3 due to releasing activity must be from unacetylated Smc3 molecules.

Because most cohesin in G2/M is associated with chromosomes, NScc1 disengagement upon TEV cleavage could take place either shortly before or after cleaved complexes are released from chromatin. Indeed, if transient disengagement drives cohesin's release from chromatin, then it must take place within complexes associated with chromatin. To address whether releasing activity also acts on cohesin whose cleavage had previously triggered dissociation from chromatin, we used BMOE-induced crosslinking to address the fate of separase-created 1–181 NScc1 in G2/M phase cells whose *WPL1* gene is under control of the galactose-inducible *GAL1-10* promoter (*GAL-WPL1*). In G2/M phase-arrested cells grown in the absence of galactose (i.e., without Wapl), 1–181 NScc1 accumulates and can be efficiently crosslinked to Smc3. Importantly, live imaging demonstrated that activation of separase at the metaphase to anaphase transition induces cohesin's removal from chromatin even in the absence of Wapl (data not shown), so that the cohesin complexes containing NScc1 (but not CScc1, which will have been degraded by Ubr1) will be nucleoplasmic and not associated with chromatin.

Induction of Wapl by galactose had no effect on crosslinking between Smc3 S1043C and C56 within FL Scc1 but greatly reduced it within 1–181 NScc1 (Figure 2D). The amount of separase-generated 1–181 NScc1 immunoprecipated with Smc3 in the absence BMOE also declined upon Wapl induction (Figure S1B). We conclude that Wapl promotes disengagement of NScc1 from Smc3 in soluble cohesin complexes that in all probability lack Scc1's C-terminal cleavage fragment. A corollary is that although releasing activity requires Scc3, it does not require the latter's association with its known Scc1 binding site, which will have been destroyed by Ubr1 (Roig et al., 2014).

Imaging Wapl-Dependent NScc1 Dissociation

To observe disengagement in living cells, we integrated 448 tetracycline operators (TetO) between the *BMH1* and *PDA1* genes in haploid or diploid *WT* cells that express both Scc3

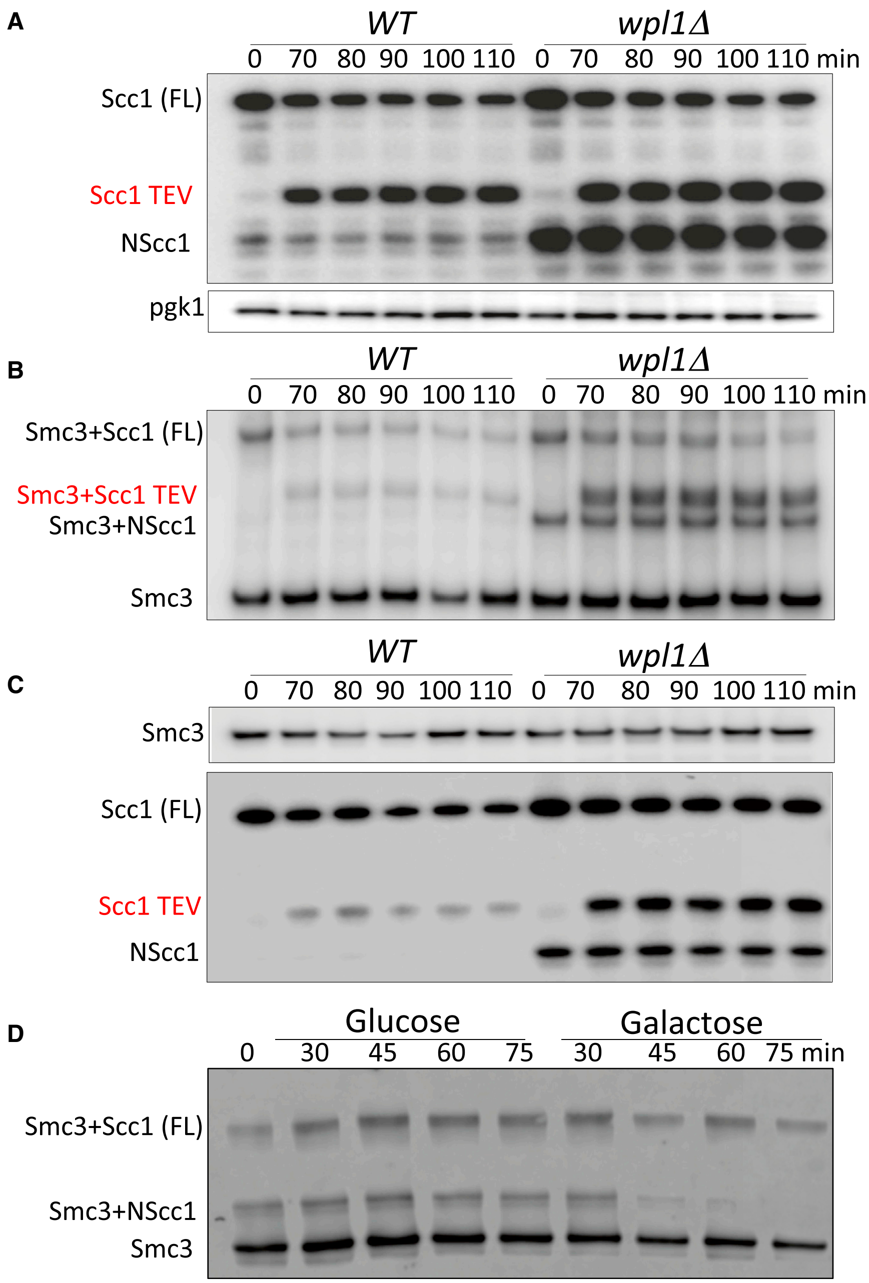

Figure 2. Wapl Triggers Dissociation of NScc1 from Smc3

(A) Wild-type and *wpl1Δ* strains K22156 (*MATα SMC3(S1043C)-HA6 MYC3-SCC1(TEV268) YEp-PGAL1 TEV*) and K22155 (*MATα wpl1Δ SMC3(S1043C)-HA6 MYC3-SCC1(TEV268) YEp-PGAL1 TEV*) grown to logarithmic phase at 25°C in YP medium containing raffinose were G2/M arrested by incubating with nocodazole for 2 hr. TEV protease was then induced by addition of galactose and cleavage of Scc1 monitored by Western blotting, detecting Myc epitopes.

(B) Samples from (A) were treated with 5 mM BMOE to induce in vivo thiol specific crosslinking between Smc3 S1043C and Scc1 C56. Smc3-HA3 immunoprecipitated from whole-cell extracts was analyzed by western blotting detecting HA epitopes.

(C) Smc3-HA3 was immunoprecipitated from untreated cells. Coimmunoprecipitation of Scc1 protein was analyzed by western blotting using Myc antibodies.

(D) Strain K22555 (*MATα SMC3(S1043C)-HA6 MYC3-SCC1(TEV268) pGAL1-10-WPL1*) expressing Wapl from the *GAL* promoter was grown in YP medium containing raffinose at 25°C and arrested in G2/M due to incubation with nocodazole for 2 hr. One-half of the culture was incubated in the presence of glucose, while the other half was induced to express Wapl by galactose addition. BMOE-induced crosslinking between Smc3 S1043C and Scc1 C56 was analyzed by western blotting using anti-Myc antibodies.

and mCherry fused to the tetracycline repressor (Scc3-TetR and TetR-mCherry). Scc1 or Smc3 tagged with GFP at their C termini expressed in this strain colocalized with TetR-mCherry, indicating that the cohesin ring is specifically tethered at the TetO arrays by Scc3-TetR, which binds to the C-terminal half of Scc1. Interestingly, in both cases enrichment of GFP at TetO arrays was detectable not only in anaphase but also in telophase cells (Figures 3A and 3B), implying that separase cleavage does not provoke immediate removal/destruction of CScc1 bound to Smc1/Smc3 heterodimers. Unlike GFP attached to Scc1's C terminus, GFP attached to its N terminus was never observed in telophase cells (Figure 3C). In contrast, it was invariably observed at the arrays in telophase *wpl1Δ* cells. We conclude that releasing activity removes most if not all NScc1 from Smc3 within 10 min of cleavage, a process more rapid than CScc1 degradation.

Scc3's Highly Conserved Surface Is Essential for Release, Not Loading

If cohesin's release from chromosomes is mediated by the transient disengagement of Scc1's NTD from Smc3, and if dissociation of NScc1 is a valid measure of the latter, then all mutations known to inactivate releasing activity and capable of suppressing null alleles of *eco1* should be defective in removing NScc1 from Smc3. We addressed first the role of cohesin's regulatory subunit Pds5. Nonlethal mutations within the N-terminal domain of Pds5, for example *pds5S81R*, enable cells to proliferate in the absence of Eco1, greatly reduce cohesin's turnover on chromosomes (Chan et al., 2012), and delay NScc1 degradation (Figure 1B). To address whether *pds5S81R* blocks its dissociation from Smc3, we analyzed the effect on BMOE-induced crosslinking between Smc3 S1043C and Scc1 C56. Figure 4C shows that the mutation greatly increases the crosslinking, implying that it does indeed delay dissociation.

We next addressed the role of Scc3. *scc3E202K* delays NScc1 degradation, suggesting that this mutation also delays NScc1's

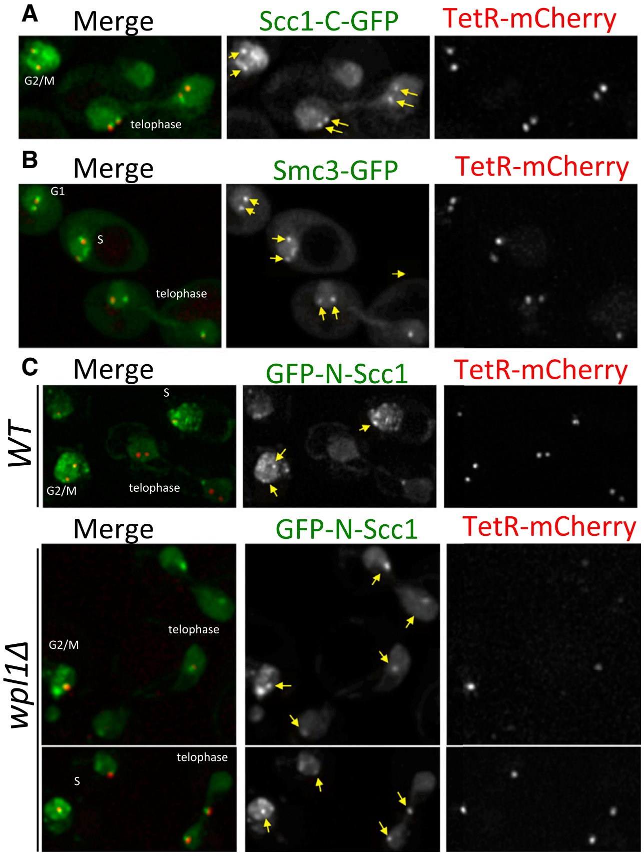

Figure 3. Live-Cell Imaging NScc1's Dissociation from Smc3

(A and B) An array of 448 tetracycline operators (TetO) was integrated between the *BMH1* and *PDA1* genes on the long arm of chromosome V in haploid (C, K23761 and K23764) or diploid (A, K23388; B, K23183) cells expressing a version of Scc3 fused to the Tet repressor as well as low levels of a Tet repressor protein fused to mCherry (TetR-mCherry) to mark the location of operators. Exponentially growing cells (in YPD medium at 25°C) were placed on 2.5% agarose pads made of synthetic complete medium containing glucose. Live-cell imaging was performed under a spinning disk confocal system at 25°C. The recruitment of C-terminally GFP-tagged Scc1 (A) and C-terminally GFP-tagged Smc3 (B) to the TetO arrays through Scc3-TetR fusion protein is shown (arrows).

(C) The localization of N-terminally GFP-tagged Scc1 to TetO arrays in wild-type (upper panel) *wapl1Δ* cells (lower panels) is marked with arrows. We failed to detect GFP-NScc1 at the Tet operators in 20 or more late anaphase/telophase nuclei in Wpl1^{+} cells.

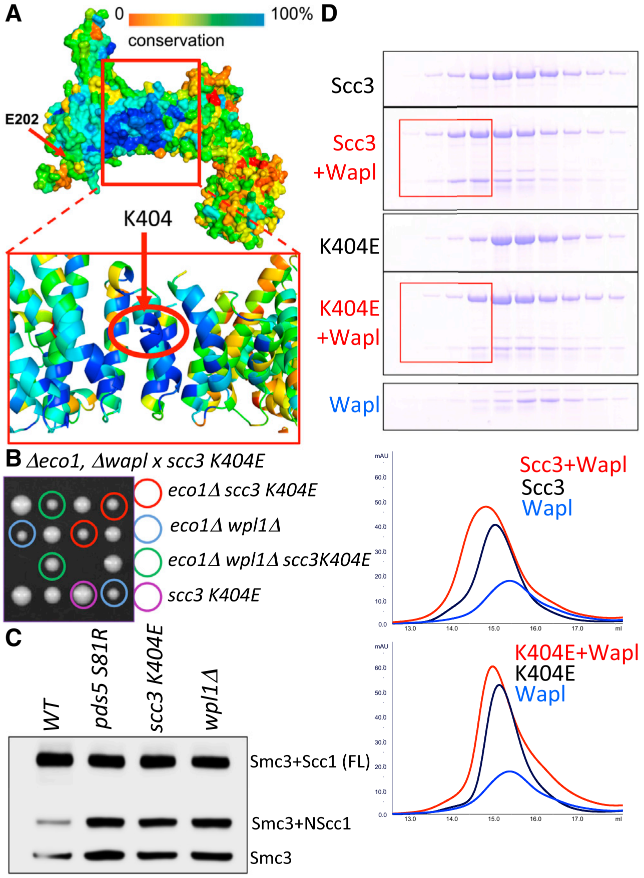

Figure 4. Scc3's Highly Conserved Surface Is Essential for NScc1 Dissociation

(A) Surface conservation of Scc3 orthologs projected on *Z.r. Scc3* (blue, most conserved; red, least conserved) highlighting the conserved K404 (*S. cerevisiae*).

(B) Diploid strain (*MATa/α wpl1Δ eco1Δ scc3K404E*) was sporulated, tetrads dissected, and selected haploid segregants with their genotypes shown.

(C) Exponentially growing cells from wild-type (K22156), *wpl1Δ* (K22155), *pds5-S81R* (K20521), and *scc3-E404K* (K24349), all *MATα SMC3(S1043C)-HA6 MYC3-SCC1(TEV268)* were grown in YPD medium at 25°C and treated with 5 mM BMOE to crosslink Smc3 S1043C and Scc1 C56. Crosslinking was analyzed by western blotting using anti HA antibody.

(D) HIS-tagged wild-type Scc3, *Scc3^{K404E}*, and Wapl proteins were purified from *E. coli* using TALON resin followed by Size exclusion using Superdex 200 16/60 column. Wild-type Scc3 or the K404E mutant protein was incubated either alone or with Wapl. After separation of the proteins by gel filtration using a Superose 6 column, the peak fractions were analyzed by SDS-PAGE and Coomassie staining, the fractions containing the Scc3/Wapl complexes are highlighted with a red box. The peak profiles of Scc3, Wapl, and the Scc3 Wapl complex are shown in the right for the wild-type and the E404K mutant proteins.

release. However, the pocket affected by this mutation is not particularly conserved, and we therefore turned our attention to the role of a highly conserved surface that sits underneath Scc3's prominent nose (Figure 4A) (Hara et al., 2014; Roig et al., 2014). Given its extreme conservation, we expected that it participates in an essential process, such as cohesin loading. Surprisingly, substitution by alanine of seven highly conserved surface residues within the domain (Scc3 K364A, Y371A, K372A, T401A, K404A, W408A, R449A −7A) is not lethal (Figures S2A and S2C). However, we observed that the 7A mutation affects releasing activity, as it permits proliferation of cells lacking Eco1 (Figure S2B). To evaluate the role of individual residues, we created a series of single mutations (K404E, K404A, Y405E, and Y405A) and used tetrad analysis to evaluate their ability to suppress lethality associated with *eco1Δ*. Of these, K404E suppressed lethality, and did so as efficiently as *waplΔ* (Figures 4B and S2B). Gel filtration showed that K404E affects binding between purified FL Scc3 and Wapl proteins (Figure 4D), though it does not eliminate the interaction. Interestingly, *scc3K404E*

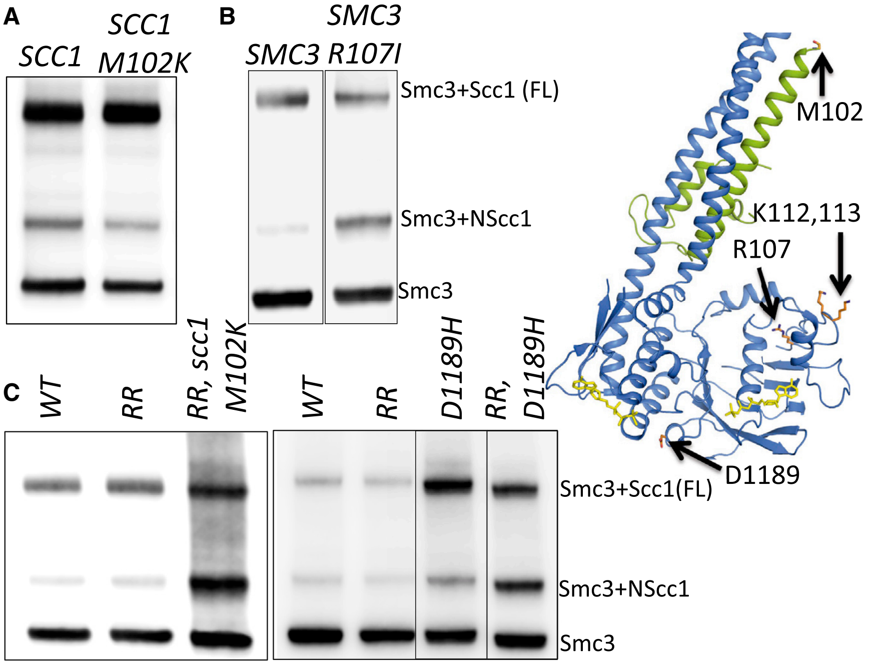

Figure 5. Effect of *smc3* and *scc1* Mutations on NScc1 Release

(A) Strains K24297 (*MATa SMC3(S1043C)-HA6 MYC3-SCC1*) and K24343 (*MATa SMC3(S1043C)-HA6 MYC3-scc1M102K*) growing exponentially in YPD medium at 25°C were treated with 5 mM BMOE to crosslink Smc3 S1043C with either wild-type Scc1 or Scc1 M102K. The crosslinking was analyzed by western blotting using an HA epitope-specific antibody. The structure of Smc3-Scc1NTD complex (PDB: 4UX3) is shown on the right with Scc1 M102, Smc3 R107, K112, K113, and D1189 residues marked.

(B) Strains K24217 (*MATa SMC3 URA3::SMC3 S1043C-PK6 MYC3-SCC1*) and K24485 (*MATa SMC3 URA3::SMC3 R107I S1043C-PK6, MYC3-SCC1*) were treated as in (A) and crosslinking analyzed by western blotting using anti PK antibody. The data are from the same western blot, with irrelevant lanes removed.

(C) Strains K24217 (*MATa SMC3 URA3::SMC3 S1043C-PK6 MYC3-SCC1*), K24493 (*MATa SMC3 URA3::smc3 K112 K113R S1043C-PK6 MYC3-SCC1*), K24495 (*MATa, SMC3, URA3::smc3 K112 K113R S1043C-PK6 MYC3-SCC1 M102K*), K24497 (*MATa SMC3 URA3::SMC3 S1043C D1189H-PK6 MYC3-SCC1*), and K24499 (*MATa SMC3 URA3::smc3 K112 K113R S1043C D1189H-PK6 MYC3-SCC1*) were analyzed as in (B). The data shown in the right panel are from the same blot, with irrelevant lanes removed.

(and the 7A mutant) does not affect accumulation of GFP tagged Wapl within pericentric chromatin in living cells (Figure S2C), implying that Wapl can still be recruited to chromosomal cohesin complexes. Crucially, *scc3K404E* caused an increase in NScc1-Smc3 crosslinking comparable to that caused by *waplΔ* and *pds5S81R* (Figure 4C). Remarkably, the very same mutation reduces binding of an N-terminal fragment of Wapl to SA2 (Scc3's mammalian ortholog) in vitro and enables mitotic cells to maintain cohesion between sister chromatids upon Sgo1 depletion (Hara et al., 2014), consistent with a releasing activity defect.

These results show that Scc3's conserved surface is essential for releasing activity and not for loading and that, like Wapl and Pds5, it has a crucial role in dissociation of NScc1 from Smc3′s coiled coil. Strangely, the part of Wapl that binds to this domain within SA2 (a small domain N terminal to its highly conserved TPR repeats) is not conserved between animal cells and fungi. However, an equivalent domain conserved among fungi exists within a similar part of fungal Wapl proteins and its deletion enables *S.cerevisiae* to proliferate in the absence of Eco1 (data not shown).

Role of Residues within Scc1's NTD

If cohesin's release from chromatin is mediated by dissociation of Scc1's NTD from Smc3, then one might expect to find mutations within the NTD that affect this process. Because no such mutations have hitherto been isolated as spontaneous *eco1* suppressors, we used mutagenic PCR and gap repair to generate a pool of mutations within an *SCC1* gene carried on a centromeric minichromosome and selected those that enable cells with a temperature sensitive allele of *eco1-1* (G211D) to form colonies at their lowest restrictive temperature 30°C. This yielded a plasmid expressing Scc1 M102K. When integrated into an ectopic locus in absence of the endogenous *SCC1*, *scc1M102K* enabled ts *eco1-1* cells to proliferate at 30°C without any accompanying increase in Smc3 acetylation (Figures S3A and S3C) and suppressed lethality caused by *smc3K112R K113R*, but not that caused by *eco1Δ*, raising the possibility that Eco1 might have an essential target in addition to Smc3's K112 and K113 residues.

An alternative explanation is that *scc1M102K* causes only a partial loss of releasing activity; that substitution of K112 and K113 by arginine also reduces releasing activity, though not below a level sufficient to restore viability; and that the combination of *smc3K112R K113R* and *scc1M102K* reduces releasing activity further, to a level compatible with proliferation in the absence of Eco1. Consistent with this scenario, *scc1M102K* suppressed lethality caused by *eco1Δ* in *smc3K112R K113R* cells (Figure S3B). Indeed, the presence or absence of *ECO1* had little or no effect on proliferation of *smc3K112R K113R scc1M102K* double mutants, suggesting that K112 and K113 may after all be the only essential targets of Eco1.

Crucially, *scc1M102K* had little or no effect on the degree of crosslinking between NScc1 and an ectopically expressed epitope tagged Smc3 S1043C protein but caused a marked increase with Smc3 S1043C K112R K113R (RR) (Figures 5A and 5C). Note that *smc3K112R K113R* also had little effect in cells containing a wild-type *SCC1* gene. These results identify mutations within the interface between Smc3 and Scc1's NTD that affect both releasing activity and dissociation of NScc1 from Smc3's coiled coil. Our findings that *scc1M102K* only causes a decrease in releasing activity sufficient to suppress *eco1Δ*

when combined with *smc3K112R K113R* and that both mutations are required to eliminate NScc1's dissociation from Smc3 further strengthens the correlation between these two phenomena and raises the possibility that NScc1 dissociation is actually responsible for cohesin's release from chromatin. How *scc1M102K* affects NScc1's dissociation from Smc3 at the hands of Wapl, Pds5, and Scc3 is presently unclear.

Role of Residues within Smc3's ATPase Domain

To probe further the relationship between releasing activity responsible for *eco1* mutant lethality and NScc1 dissociation, we measured the effect of mutations within Smc3's ATPase. Smc3 R107 is a highly conserved arginine within the upper of three parallel β sheets that underlie the loop containing K112 and K113 (Figure 5B). It faces inside the ATPase domain and mutation to either isoleucine or alanine enables robust proliferation of *eco1Δ* cells (Rowland et al., 2009). Figure 5B shows that, like *scc3*, *wpl1*, and *pds5* suppressors, *smc3R107I* also blocks NScc1 dissociation as measured by crosslinking between Smc3 S1043C and Scc1 C56.

We next analyzed the effect of an *smc3* mutation at the base of Smc3's ATPase domain (*smc3D1189H*) isolated not as an *eco1* suppressor but by virtue of its ability to suppress the sensitivity to benomyl of *wpl1Δ eco1Δ* double mutants (Guacci et al., 2015). Like *scc1M102K*, *smc3D1189H* suppresses lethality associated with *smc3K112R K113R*, but not *eco1Δ*. Figure 5C shows that *smc3D1189* causes only a modest increase in crosslinking between Smc3 S1043C and Scc1 C56 but that this effect is greatly increased when K112 and K113 are replaced by arginine. As in the case for *scc1M102K*, *smc3K112R K113R* enabled *smc3D1189H* to suppress lethality associated with *eco1Δ* (Figure S4A). The finding that *smc3D1189H* suppresses the lethality of *smc3K112R K113R* but not *eco1Δ* led to the suggestion that Eco1 must therefore have targets besides K112 and K113 (Guacci et al., 2015). Our data suggest an alternative and more plausible explanation, namely that cohesin complexes containing *smc3K112R K113R* have less releasing activity than unacetylated wild-type complexes. Thus, the properties of *smc3D1189H* do not imply that Eco1 has targets besides K112 and K113. The behavior of *smc3D1189H* reveals again an exquisite correlation between *eco1Δ* suppression and defective NScc1 dissociation. These findings have an interesting corollary. If one assumes that rescue by *smc3D1189H* of the benomyl sensitivity of *wpl1Δ eco1Δ* double mutants (Guacci et al., 2015) is due to a defect in releasing activity, then residual releasing activity must persist in *wpl1Δ* mutants.

Smc3 Acetylation Blocks NScc1 Dissociation

To address whether Smc3 acetylation blocks disengagement of Scc1's NTD from Smc3, we asked whether the decline in crosslinking between NScc1 and Smc3 S1043C upon induction of Wapl from *GAL-WPL1* depends on Smc3's deacetylation by Hos1. Figure 6A shows the decline is less pronounced in *hos1Δ* cells than in wild-type. In other words, NScc1's dissociation upon Wapl reactivation depends on the latter's prior deacetylation at the time of separase cleavage. The failure to remove NScc1 from Smc3 in *hos1* mutant cells might contribute to the inability of Smc3 molecules that remain acetylated after anaphase to build cohesion during the subsequent cell cycle (Beckouët et al., 2010).

Another way of addressing whether acetylation blocks disengagement of NScc1 from Smc3 is to analyze the effect of mutations such as *smc3K112Q K113Q* that are thought to mimic acetylation. It has hitherto not been possible to address the effect of *smc3K112Q K113Q* on releasing activity directly, because the mutation causes a major decline in cohesin's loading throughout the genome (Hu et al., 2015). Without loading, it is not possible to measure release. Our finding that NScc1 dissociation appears to be a perfect surrogate for release solves this conundrum. All that is required is that Scc1 associated with Smc3 K112Q K113Q be cleaved by separase. Fortunately, this proves to be the case, and Figure 6B shows that *smc3K112Q K113Q* elevates crosslinking between NScc1 and Smc3S1043C to a degree comparable to *wpl1Δ*.

Disengagement of NScc1 from Smc3 Involves Engagement of Smc ATPase Heads

To address the role of ATP hydrolysis, we used BMOE-induced crosslinking between Smc3 S1043C and Scc1 C56 to measure the effect of Smc3 E1155Q (Arumugam et al., 2003) on dissociation from Smc3 of NScc1 created in nocodazole-arrested cells by TEV protease. Figure 7A shows that crosslinking between Scc1 C56 and Smc3 S1043C is little affected by Smc3E1155Q, either when NScc1 is created by separase during the previous cell cycle or by TEV protease during nocodazole-induced G2/M phase arrest. This implies that the EQ mutation does not prevent dissociation. To confirm this, we compared the effect of inducing Wapl in *GAL-WPL1 eco1Δ* cells on BMOE-induced crosslinking between NScc1's C56 and Smc3 S1043C and Smc3 E1155Q S1043C. Interestingly, the *smc3E1155Q* mutation again had little or no effect (Figure S4B). Further confirmation that releasing activity dissociates NScc1 from Smc3 E1155Q ATPases is our finding that *smc3K112Q K113Q* causes a significant increase in NScc1 C56 crosslinking to Smc3 E1155Q S1043C (Figure 7B), suggesting that the dissociation reaction in Smc3 E1155Q complexes is affected by acetylation of K112 and K113.

We were unable to address the role of ATPase head engagement by analyzing mutations like *smc3K39I*, which affects binding of ATP to the Smc3 ATPase heads, or the signature motif mutation *smc3S1128R*, which compromises engagement with Smc1 ATPase heads (Arumugam et al., 2006), because neither Smc3 K39I nor Smc3 S1128R proteins accumulate within nuclei (data not shown) and may not even interact with Wapl, which is a nuclear protein. Nevertheless, in the course of our studies, a novel class of *eco1Δ* suppressor mutations has been discovered, namely *smc1D1164E*, which alters a key amino acid within the Smc1 ATPase's D loop, and *smc1L1129V*, which alters its signature motif (Figure S4C). These mutations cause a severe reduction in ATPase activity (without affecting ATP binding) but cause only a mild reduction in the amount of cohesin loaded throughout the genome (Elbatsh et al., 2016, in this issue of *Molecular Cell*). To test whether *smc1D1164E* or *smc1L1129* affects dissociation of NScc1 from Smc3, we measured their effects on crosslinking between Smc3 S1043C and C56 from a version of Scc1 whose N terminus was tagged with multiple

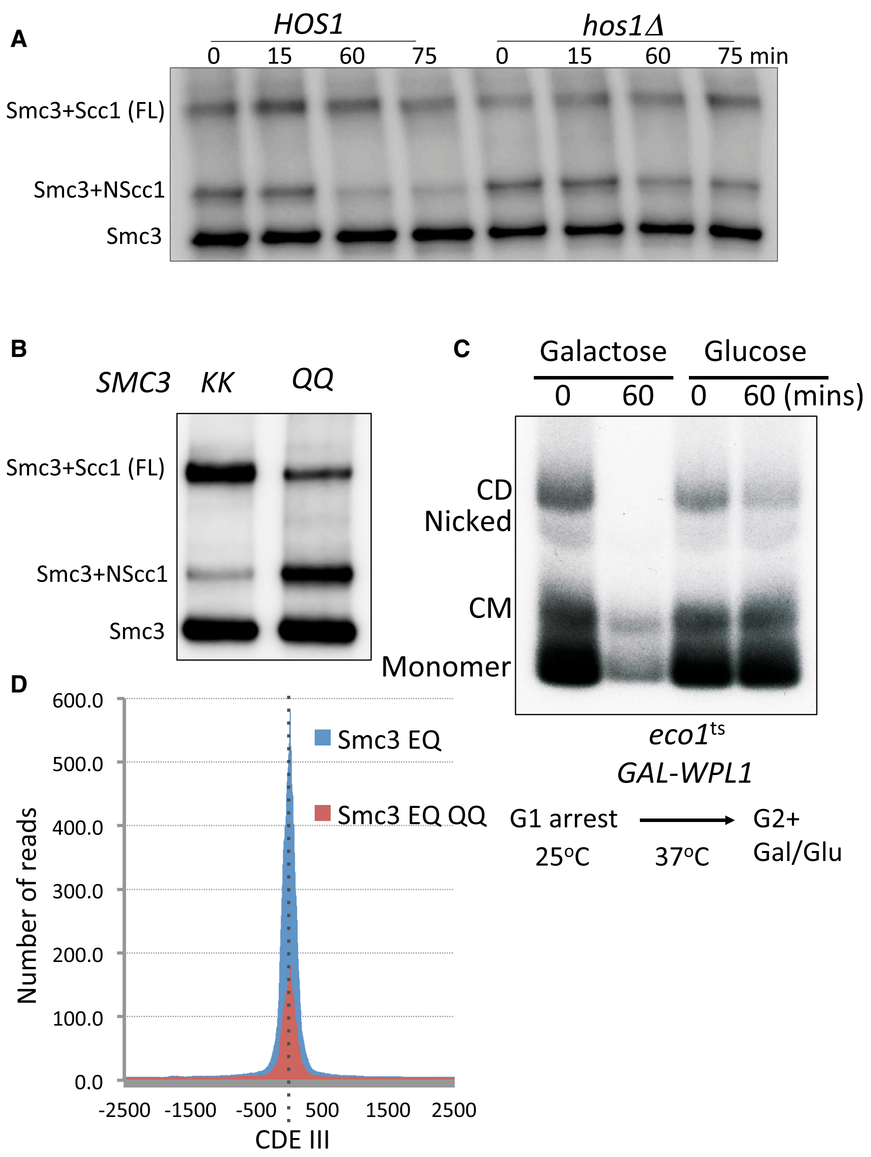

Figure 6. Smc3 Acetylation Blocks NScc1 Dissociation

(A) *HOS1* or *hos1Δ* strains with galactose inducible *WPL1*, K22555 (*MATa SMC3 (S1043C)-HA6 MYC3-SCC1(TEV268) pGAL1-10-WPL1*) and K22810 (*MATa hos1Δ SMC3(S1043C)-HA6 MYC3-SCC1(TEV268) pGAL1-10-WPL1*) were grown in YP Raff medium at 25°C and arrested in nocodazole for 2 hr. Galactose was then added to induce Wapl. Samples were taken at the indicated time points to induce in vivo crosslinking with 5 mM BMOE. Crosslinking was analyzed by western blotting using anti HA antibodies. Uncrosslinked samples were also analyzed similarly (shown in Figure S1B).

(B) Exponentially growing strains K24217 (*MATa SMC3 URA3::SMC3 S1043C-PK6 MYC3-SCC1(TEV268)*) and K24218 (*MATa, SMC3 URA3::smc3K112 113Q S1043C-PK6 MYC3-SCC1 (TEV268)*) in YPD medium at 25°C were subjected to in vivo thiol-specific crosslinking with 5 mM BMOE. Crosslinking was analyzed by western blotting using anti PK(V5) antibody.

(C) Strain K24090 containing a 2.3 kb circular mini-chromosome, six cysteines within the Smc1-Smc3-Scc1 interfaces, *eco1^{ts}(G211H)*, and galactose-inducible WPL1 gene was grown at 25°C, arrested in G1 by pheromone, and permitted to go through S phase at 37°C in the presence of nocodazole. After addition of either galactose or glucose to induce Wapl expression (or not) samples were taken at times 0 and 60 min for in vivo crosslinking with BMOE. Scc1-PK6-immunoprecipitated DNA denatured with SDS was detected by Southern blotting. Catenated monomers (CM), catenated dimers (CD).

(D) Calibrated ChIP-seq profiles of Smc3 E1155Q and Smc3 E1155Q K112Q K113Q showing the number of reads at each base pair away from the CDEIII element averaged over all 16 chromosomes.

myc epitopes. We used this method instead of using an epitope tag on Smc3's C terminus (as performed in all previous experiments), because tetrad analysis revealed that the latter was synthetic lethal with both *smc1D1164E* and *smc1L1129V* (data not shown). As shown in Figures 7C and S4C, both mutations increased crosslinking between NScc1 and Smc3.

The equivalent residues within Smc3 are D1161 and L1126. *smc3L1126V* is viable despite reducing ATPase activity and cohesin's association with chromosomes, but it fails to suppress the temperature sensitivity of an *eco1-1* strain (Elbatsh et al., 2016) and has no effect on crosslinking between Smc3 S1043C and Scc1C56 (Figure 7C). *smc3D1161E* is lethal, and the protein fails to enter nuclei, precluding analysis of its effect on releasing activity or *eco1-1* suppression (Elbatsh et al., 2016).

These results reveal that a surface on Smc1's ATPase that interacts with ATP sandwiched between Smc1 and Smc3 heads is crucial for NScc1 release. The different behavior with regard to NScc1 dissociation of *smc1D1164E* or *smc1L1129V* and *smc3E1155Q* suggests that the former mutations affect a different step in the ATP hydrolysis cycle than the latter. The lack of any effect of *smc3E1155Q* on NScc1 dissociation implies that only part of a single ATP hydrolysis cycle is necessary. Though ATP hydrolysis per se is not required, an early event following the cooperative binding of ATP to Smc heads appears necessary. Abolition of releasing activity by *smc1L1129V*, but not by *smc3L1126V*, a pair of mutations that have equally severe effects on ATP hydrolysis and cohesin loading, suggests that the nature of ATPase head engagement required for release differs fundamentally from that required for loading. There may be more than one state of head engagement.

Though our crosslinking assay is designed to measure NScc1 dissociation, it also reveals crosslinking between Smc3 S1043C and C56 from intact Scc1 molecules. We noticed that several mutations that affect ATP hydrolysis, in particular *smc3E1155Q* and possibly also *smc3D1189H*, appear to increase the efficiency of Scc1-Smc3 crosslinking. We do not understand the significance of this effect. Crucially, it does not correlate with

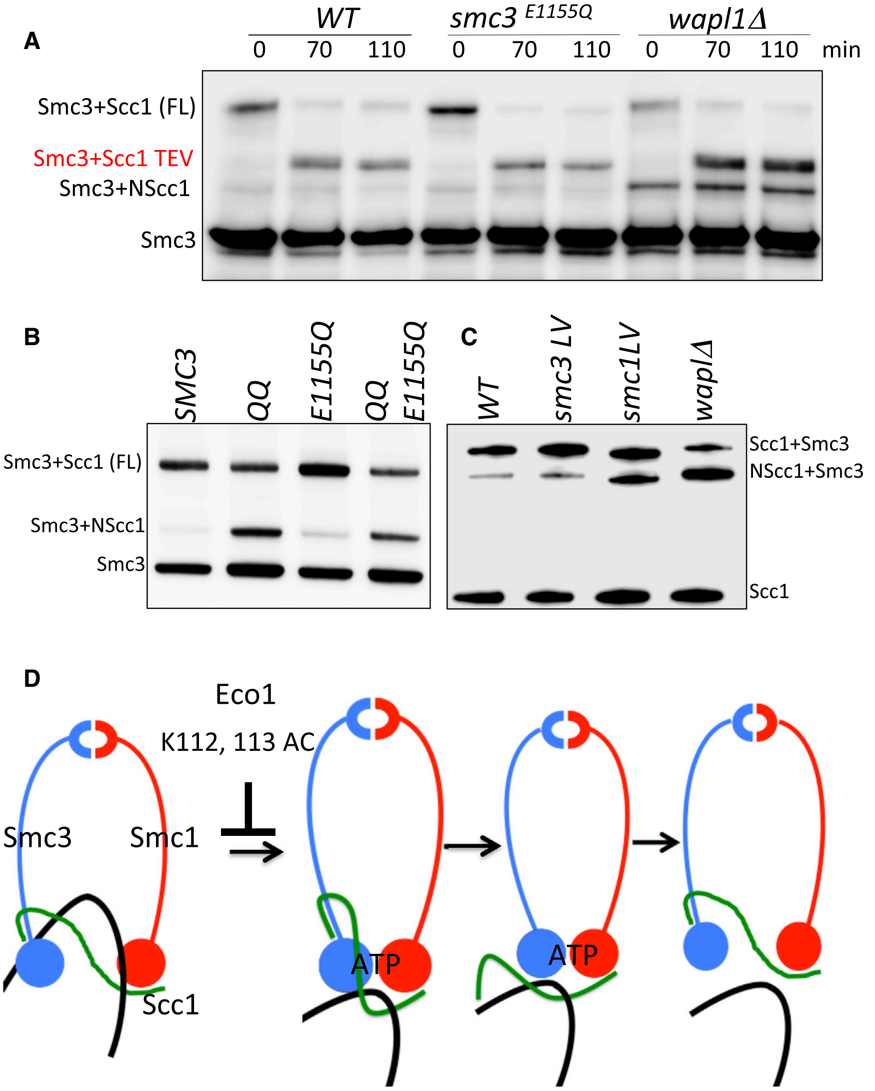

Figure 7. Disengagement of NScc1 from Smc3 Requires a Single Round of ATP Hydrolysis

(A) Strains K23070 (*MATa SMC3 URA3::SMC3 S1043C-HA6 MYC3-SCC1(TEV268)* YEp-PGAL1-TEV), K23067 (*MATa SMC3 URA3::smc3 S1043C E1155Q-HA6::URA3 MYC3-SCC1(TEV268)* YEp-PGAL1-TEV), and K23068 (*MATa SMC3 SMC3 S1043C-HA6 MYC3-SCC1(TEV268) wpl1Δ* YEp-PGAL1 TEV) were treated and analyzed as described in Figure 2B.

(B) Strains K24217 (*MATa SMC3 URA3::SMC3 S1043C-PK6::URA3 MYC3-SCC1(TEV268)*), K24218 (*MATa SMC3 URA3::smc3K112 K113Q S1043C-PK6 MYC3-SCC1(TEV268)*), K24219 (*MATa SMC3 URA3::smc3E1155Q S1043C-PK6 MYC3-SCC1(TEV268)*), and K24220 (*MATa SMC3 URA3::smc3K112 K113Q E1155Q S1043C-PK6 MYC3-SCC1(TEV268)*) were grown and analyzed as described in Figure 6B.

(C) Exponentially growing strains K23070, K23068, K24911 (*SMC3 smc3L1126V S1043C::LEU2 MYC3-SCC1(TEV268)*), and K24523 (*SMC3 S1043C::ADE2 MYC3-SCC1(TEV268) smc1 L1129V*) were treated as described in Figure 6B and analyzed by western blotting using anti-MYC antibodies.

(D) Shown is a model for how releasing activity dissociates Scc1-NTD from Smc3's coiled coil leading to escape of entrapped DNAs in a process involving ATP-dependent engagement of SMC ATPase heads. Acetylation of Smc3 residues K112 and K113 is suggested to inhibit ATP-dependent head engagement.

defects in NScc1 dissociation, and we therefore presume that it is immaterial to our measurements of NScc1 cleavage fragment crosslinking.

The Mechanism by which Acetylation Blocks Release

The finding that NScc1 release depends on the precise form of interaction between Smc1 and Smc3 ATPase heads raises the possibility that acetylation blocks release by regulating this process. For the lack of an assay to measure directly the state of interaction between Smc1 and Smc3 ATPase heads in vivo, we investigated whether the chemical status of K112 and K113 affects the behavior of Smc3 E1155Q cohesin complexes. The inability of these complexes to hydrolyze ATP stabilizes head engagement and prevents cohesin's translocation into pericentric sequences from its loading sites at core centromeres (Hu et al., 2011, 2015). These complexes are nevertheless capable of releasing NScc1 from Smc3. If acetylation blocked a step prior to that blocked by E1155Q, for example a certain type of head engagement, then K112Q K113Q should block the ability of Smc E1155Q to associate with centromeres. Calibrated ChIP-seq profiles of Smc3 E1155Q and Smc3 E1155Q K112Q K113Q, averaged over all 16 centromeres, shows that K112Q K113Q reduces association of Smc3E1155Q, at least 3-fold (Figure 6D). This suggests that acetylation blocks the Smc1/3 head engagement necessary for association of Smc3E1155Q cohesin with its centromeric loading sites, raising the possibility that it blocks release via a related mechanism.

Releasing Activity Induces Exit of DNAs from Cohesin Rings

To prove that releasing activity does actually induce DNAs to escape entrapment by cohesin rings, we created an *eco1-1(G211H) GAL-WPL1* yeast strain with cysteine pairs at all three interfaces (hence 6C) making up the cohesin ring (Gligoris et al., 2014). Cells (containing a circular 2.5 kb minichromosome) growing at 25°C were arrested in G1 by pheromone and then permitted to undergo S phase at 37°C (the restrictive temperature for *eco1-1(G211H)*) in the presence of nocodazole. The G2/M-arrested cells were treated with BMOE and DNAs associated with 6C crosslinked cohesin immune-precipitated, heated in the presence of SDS at 65°C before gel electrophoresis, and minichromosome DNA detected by Southern blotting. The

absence of Wapl permits such cells to build cohesion, and this is apparent in the formation of sister DNAs catenated by cohesin rings containing BMOE-induced crosslinks at all three interfaces (catenated dimers or CDs). Monomeric DNAs catenated by covalently closed cohesin rings (CMs) are also apparent. Crucially, addition of galactose to the G2/M phase cells, which restores Wapl to cells lacking Smc3 acetylation, causes a rapid decline of both CDs and CMs as well as the amount of monomeric DNAs merely coprecipitated with cohesin (Figure 6C). A modest decline in CDs, but not CMs, was also observed in the absence of galactose, possibly due to the extended incubation at 37°C. We conclude that releasing activity does indeed catalyze escape of DNAs from inside cohesin's ring.

DISCUSSION

The kinetics of cohesin's association with chromosomes is determined by loading mediated by the Scc2/4 kollerin complex and release mediated by cohesin's Wapl, Pds5, and Scc3 subunits. If loading involves entrapment of DNAs within cohesin's ring, then release must involve their subsequent escape through an exit gate. We describe here definitive evidence that DNAs are indeed induced to escape in this manner in living cells (Figure 7D). The observation that cohesin containing an Smc3-Scc1 fusion protein loads but cannot dissociate suggests that the exit gate is situated at the interface between Scc1's NTD and the coiled coil emerging from Smc3's ATPase. However, this is not proof. Fusion of Smc3 to Scc1 could conceivably block release not by creating a topological barrier to DNA exit but instead by altering the ATPase's conformation, which could have an indirect effect on release via an exit gate situated elsewhere. It was vital, therefore, to address whether or not cohesin's releasing activity does indeed disengage Scc1's NTD from Smc3.

The technology we adopted is chemical crosslinking induced by the bifunctional thiol-specific reagent BMOE, which induces within minutes efficient crosslinks between Smc3 S1043C within Smc3's coiled coil and a natural cysteine (C56) in Scc1's NTD. We suspect that our failure to discern any effect of releasing activity on the efficiency of crosslinking within intact cohesin complexes can be attributed to release being a transient process. When Scc1 NTDs reassociate with Smc3, they will be again subject to crosslinking, which will subsequently trap them in the associated state. Our discovery that degradation of Scc1's N-terminal separase cleavage fragment is largely abolished by mutations that inactivate releasing activity provided a solution to this conundrum. It led to the realization that dissociation could be made irreversible and hence observable by measuring crosslinking between Smc3's coiled coil and N-terminal Scc1 cleavage fragments (NScc1), be they created naturally during anaphase by separase or artificially by inducing cleavage with TEV protease.

Using this assay, we have analyzed a wide variety of mutations known to have severe defects in releasing activity (because they permit proliferation in the absence of Eco1). Some of these mutations have been described previously while others are described in this or the accompanying paper (Elbatsh et al., 2016). In every single case where mutations suppress *eco1Δ*, they also reduce NScc1 dissociation. Striking examples are *scc1M102K* and *smc3D1189H*, which only suppress *eco1Δ* when the residues within Smc3's ATPase domain (K112 and K113) that must normally be acetylated by Eco1 are replaced by arginine. *Smc3K112R K113R* not only prevents acetylation but also compromises releasing activity, not to an extent that avoids lethality, but to an extent that exacerbates defects caused by *scc1M102K* and *smc3D1189H.*

The strength of this correlation means that it is hard to avoid the conclusion that releasing activity does indeed induce dissociation of the interface between Scc1's NTD from Smc3's coiled coil. The previous finding that fusion of Smc3's C terminus to Scc1's N terminus also abrogates releasing activity and suppresses *eco1Δ* lethality implies that dissociation of Scc1 from Smc3 is not only an intrinsic aspect of releasing activity but is also required for this phenomenon. Both sets of observations are consistent with the notion that cohesin loading involves entrapment of DNA inside cohesin rings and that release from chromosomes is mediated by their escape through a gate created by dissociation of Scc1's NTD from Smc3's coiled coil.

Our conclusion that cohesin's release from chromosomes is mediated by dissociation of NScc1 from Smc3's coiled coil has an important ramification. Hitherto, it has not been possible to measure directly the effect of K112 and K113 acetylation by Eco1 on release, as modification of these residues interferes with loading as well as release. Our finding that releasing activity-dependent NScc1 dissociation can also be observed within cohesin complexes that cannot load onto chromosomes means that it has been possible to measure the effect of *smc3K112Q K113Q*, which is presumed to mimic the acetylated state. Crucially, this double mutant appears to abrogate dissociation of NScc1 from Smc3 as severely as all known releasing activity mutations, implying that K112 and K113 acetylation does indeed neutralize releasing activity, as has long been suspected. Our finding that the Hos1 deacetylase is important for NScc1's dissociation from Smc3 during anaphase confirms that this conclusion applies equally well to physiological acetylation.

The mechanism by which Scc1's N-terminal domain is dissociated from Smc3's coiled coil is clearly a complex process, as it depends on Wapl, Pds5, Scc3, residues throughout Smc3's ATPase head; as well as the KKD loop that is the target of Eco1; and on residues within the D loop and signature motif of Smc1's ATPase head. The latter is a crucial discovery, as it implies that ATP-driven engagement of Smc1 and Smc3 ATPase heads is involved. In this regard, the process shares properties with the cohesin loading reaction. Indeed, it is tempting to speculate that acetylation blocks both loading and release, because it blocks ATPase head engagement, a proposal consistent with our finding that *smc3K112Q K113Q* greatly reduces association of Smc3E1155Q with centromeric loading sites. We have previously suggested that the acetylation state of the KKD loop is communicated to Smc3's ATP binding pocket via R61, which sits on top of a short α helix connecting these structures. Smc3 R61Q is lethal and compromises cohesin loading, and it will be interesting to establish whether the mutation also abolishes releasing activity. We acknowledge that the lack of any effect of *smc3K105Q K106Q* mutations on the ATPase activity of purified human trimeric Smc1/Smc3/Scc1 complexes (Ladurner

et al., 2014) appears contrary to our proposal that acetylation affects the state of head engagement. We note, however, that these assays were performed in the absence of Scc3, Pds5, or Wapl, which might alter the properties of cohsesin's ATPase heads. Moreover, the role of acetylation of vertebrate cohesin is presently unclear. While acetylation is a prerequisite for Sororin association, it is not sufficient to counteract releasing activity (which requires Sororin association to acetylated cohesin). It is therefore conceivable that any effect on the ATP hydrolysis cycle might manifest only after Sororin association.

It is nevertheless important to point out that loading is not greatly affected by *wpl1*, *pds5*, *scc3*, and *smc3* mutations that greatly reduce release. Likewise, fusion of Smc3 to Scc1, which abrogates release, still permits cohesin loading not only in yeast (Gruber et al., 2006; Haering et al., 2008) but also in *Drosophila* (Eichinger et al., 2013). Thus, while opening of the Scc1-Smc3 interface is an obligate aspect of release, it is not an obligate aspect of loading, a fact difficult if not impossible to reconcile with the suggestion that this same interface is also cohesin's main DNA entry gate (Murayama and Uhlmann, 2015). Indeed, there is evidence that cohesin's DNA entry gate is situated instead at the Smc1/Smc3 hinge interface (Gruber et al., 2006). We conclude that though release and loading share certain properties, such as involvement of an intermediate involving head engagement, they are nevertheless distinct processes with very different outcomes for the state of cohesin's association with chromatin. We speculate that release involves an intermediate in which DNA previously trapped inside heterotrimeric rings is ejected from the lumen created by engagement of Smc1 and Smc3 ATPase heads, permitting its escape when this subsequently triggers disengagement of the Scc1-Smc3 interface (Figure 7D).

There is now incontrovertible evidence that the sister DNAs of circular minichromosomes can be entrapped by heterotrimeric cohesin rings, a phenomenon that is currently the only plausible explanation for how cohesin holds sister chromatids together. How DNAs enter cohesin rings remains very unclear. This paper has revealed a plausible pathway for its subsequent exit, namely through a gate created by disengagement of the Scc1-Smc3 interface. The existence of this pathway constitutes therefore an important endorsement of the notion that cohesin and its relatives do indeed act as topological devices, as originally proposed by the ring model. Lastly, our finding that releasing activity opens the interface between Scc1 and Smc3, with the implication that this creates a gate for DNA to exit cohesin rings, suggests that topological entrapment is a universal mechanism for cohesin's stable or semistable association with chromatin fibers and not merely one that applies to minichromosomes, which hitherto is the only instance where this has been directly demonstrated.

EXPERIMENTAL PROCEDURES

Yeast Cell Culture

All strains are derivatives of W303 (K699). Strain numbers and relevant genotypes of the strains used are listed in the figure legends. Details of strain construction are in Supplemental Experimental Procedures. Cells were cultured at 25°C in YEP medium with 2% glucose unless stated otherwise.

Sequence Alignment

For the multiple alignment conservation, the following sequences were included: *Zygosaccharomyces rouxii* (C5DWM3), *Saccharomyces cerevisiae* (P40541), *Ashbya gossypii* (M9MYD6), *Homo sapiens* (Q6P275), *Xenopus laevis* (Q9DGN1), *Danio rerio* (B0V0X2), *Drosophila melanogaster* (Q9VM62), *Daphnia pulex* (E9FY68), *Brugia malayi* (A8QED2), *Vitis vinifera* (D7TP60), *Candida albicans* (C4YFQ5), *Schizosaccharomyces pombe* (O13816), and *Sordaria macrospora* (F7W0E2).

In Vivo Chemical Crosslinking and Minichromosome IP

In vivo crosslinking and minichromosome IP were performed as described by Gligoris et al. (2014) and as detailed in the Supplemental Experimental Procedures.

Live-Cell Imaging

Exponentially growing cells were placed on 2.5% agarose pads made of synthetic complete medium plus glucose. Live-cell imaging was performed under a spinning disk confocal system (PerkinElmer UltraVIEW) with an EMCCD camera (Hamamatsu) mounted on an Olympus IX81 microscope with Olympus 100× 1.35N.A. objectives. Image acquisition was done at 25°C. Seventeen to twenty-one z-stacking images were acquired, and image deconvolution was done by using Volocity software with seven iterations and 95% confidence. Fresh samples were prepared every 10 min.

Calibrated ChIP-Seqencing

Calibrated ChIP-seq was performed as detailed in (Hu et al., 2015).

ACCESSION NUMBERS

The sequencing data have been deposited in GEO with the accession number GSE76890.

SUPPLEMENTAL INFORMATION

Supplemental Information includes four figures and Supplemental Experimental Procedures and can be found with this article at http://dx.doi.org/10.1016/j.molcel.2016.01.026.

AUTHOR CONTRIBUTIONS

F.B., M.S., M.B.R., and K.-L.C. designed and conducted the experiments. J.C.S., P.B., B.H., N.P., T.G., A.C.S., and L.S. conducted the experiments. K.N. designed the experiments and wrote the paper.

ACKNOWLEDGMENTS

We are grateful to K. Shirahige for supplying anti-acetylated Smc3 antibody and to all members of the Nasmyth group for valuable discussions. This work was funded by the Wellcome Trust (091859/Z/10/Z to K.N.) and Cancer Research UK (C573/A 12386 to K.N.).

Received: July 27, 2015
Revised: November 27, 2015
Accepted: January 22, 2016
Published: February 18, 2016

REFERENCES

Arumugam, P., Gruber, S., Tanaka, K., Haering, C.H., Mechtler, K., and Nasmyth, K. (2003). ATP hydrolysis is required for cohesin's association with chromosomes. Curr. Biol. *13*, 1941–1953.

Arumugam, P., Nishino, T., Haering, C.H., Gruber, S., and Nasmyth, K. (2006). Cohesin's ATPase activity is stimulated by the C-terminal Winged-Helix domain of its kleisin subunit. Curr. Biol. *16*, 1998–2008.

Beckouët, F., Hu, B., Roig, M.B., Sutani, T., Komata, M., Uluocak, P., Katis, V.L., Shirahige, K., and Nasmyth, K. (2010). An Smc3 acetylation cycle is essential for establishment of sister chromatid cohesion. Mol. Cell *39*, 689–699.

Bürmann, F., Shin, H.C., Basquin, J., Soh, Y.M., Giménez-Oya, V., Kim, Y.G., Oh, B.H., and Gruber, S. (2013). An asymmetric SMC-kleisin bridge in prokaryotic condensin. Nat. Struct. Mol. Biol. *20*, 371–379.

Chan, K.L., Roig, M.B., Hu, B., Beckouët, F., Metson, J., and Nasmyth, K. (2012). Cohesin's DNA exit gate is distinct from its entrance gate and is regulated by acetylation. Cell *150*, 961–974.

Chan, K.L., Gligoris, T., Upcher, W., Kato, Y., Shirahige, K., Nasmyth, K., and Beckouët, F. (2013). Pds5 promotes and protects cohesin acetylation. Proc. Natl. Acad. Sci. USA *110*, 13020–13025.

Ciosk, R., Shirayama, M., Shevchenko, A., Tanaka, T., Toth, A., Shevchenko, A., and Nasmyth, K. (2000). Cohesin's binding to chromosomes depends on a separate complex consisting of Scc2 and Scc4 proteins. Mol. Cell *5*, 243–254.

De, K., Sterle, L., Krueger, L., Yang, X., and Makaroff, C.A. (2014). Arabidopsis thaliana WAPL is essential for the prophase removal of cohesin during meiosis. PLoS Genet. *10*, e1004497.

Deardorff, M.A., Bando, M., Nakato, R., Watrin, E., Itoh, T., Minamino, M., Saitoh, K., Komata, M., Katou, Y., Clark, D., et al. (2012). HDAC8 mutations in Cornelia de Lange syndrome affect the cohesin acetylation cycle. Nature *489*, 313–317.

Eichinger, C.S., Kurze, A., Oliveira, R.A., and Nasmyth, K. (2013). Disengaging the Smc3/kleisin interface releases cohesin from Drosophila chromosomes during interphase and mitosis. EMBO J. *32*, 656–665.

Elbatsh, A.M.O., Haarhuis, J.H.I., Petela, N., Chapard, C., Fish, A., Celie, P.H., Stadnik, M., Ristic, D., Wyman, C., Medema, R.H., Nasmyth, K., and Rowland, B.D. (2016). Cohesin releases DNA through asymmetric ATPase-driven ring opening. Mol. Cell *61*, this issue, 575–588.

Gandhi, R., Gillespie, P.J., and Hirano, T. (2006). Human Wapl is a cohesin-binding protein that promotes sister-chromatid resolution in mitotic prophase. Curr. Biol. *16*, 2406–2417.

Gerlich, D., Koch, B., Dupeux, F., Peters, J.M., and Ellenberg, J. (2006). Live-cell imaging reveals a stable cohesin-chromatin interaction after but not before DNA replication. Curr. Biol. *16*, 1571–1578.

Gligoris, T.G., Scheinost, J.C., Bürmann, F., Petela, N., Chan, K.L., Uluocak, P., Beckouët, F., Gruber, S., Nasmyth, K., and Löwe, J. (2014). Closing the cohesin ring: structure and function of its Smc3-kleisin interface. Science *346*, 963–967.

Gruber, S., Haering, C.H., and Nasmyth, K. (2003). Chromosomal cohesin forms a ring. Cell *112*, 765–777.

Gruber, S., Arumugam, P., Katou, Y., Kuglitsch, D., Helmhart, W., Shirahige, K., and Nasmyth, K. (2006). Evidence that loading of cohesin onto chromosomes involves opening of its SMC hinge. Cell *127*, 523–537.

Guacci, V., Koshland, D., and Strunnikov, A. (1997). A direct link between sister chromatid cohesion and chromosome condensation revealed through the analysis of MCD1 in S. cerevisiae. Cell *91*, 47–57.

Guacci, V., Stricklin, J., Bloom, M.S., Guō, X., Bhatter, M., and Koshland, D. (2015). A novel mechanism for the establishment of sister chromatid cohesion by the ECO1 acetyltransferase. Mol. Biol. Cell *26*, 117–133.

Haering, C.H., Löwe, J., Hochwagen, A., and Nasmyth, K. (2002). Molecular architecture of SMC proteins and the yeast cohesin complex. Mol. Cell *9*, 773–788.

Haering, C.H., Farcas, A.M., Arumugam, P., Metson, J., and Nasmyth, K. (2008). The cohesin ring concatenates sister DNA molecules. Nature *454*, 297–301.

Hara, K., Zheng, G., Qu, Q., Liu, H., Ouyang, Z., Chen, Z., Tomchick, D.R., and Yu, H. (2014). Structure of cohesin subcomplex pinpoints direct shugoshin-Wapl antagonism in centromeric cohesion. Nat. Struct. Mol. Biol. *21*, 864–870.

Hu, B., Itoh, T., Mishra, A., Katoh, Y., Chan, K.L., Upcher, W., Godlee, C., Roig, M.B., Shirahige, K., and Nasmyth, K. (2011). ATP hydrolysis is required for relocating cohesin from sites occupied by its Scc2/4 loading complex. Curr. Biol. *21*, 12–24.

Hu, B., Petela, N., Kurze, A., Chan, K.L., Chapard, C., and Nasmyth, K. (2015). Biological chromodynamics: a general method for measuring protein occupancy across the genome by calibrating ChIP-seq. Nucleic Acids Res. *43*, e132.

Ivanov, D., Schleiffer, A., Eisenhaber, F., Mechtler, K., Haering, C.H., and Nasmyth, K. (2002). Eco1 is a novel acetyltransferase that can acetylate proteins involved in cohesion. Curr. Biol. *12*, 323–328.

Kueng, S., Hegemann, B., Peters, B.H., Lipp, J.J., Schleiffer, A., Mechtler, K., and Peters, J.M. (2006). Wapl controls the dynamic association of cohesin with chromatin. Cell *127*, 955–967.

Ladurner, R., Bhaskara, V., Huis in 't Veld, P.J., Davidson, I.F., Kreidl, E., Petzold, G., and Peters, J.M. (2014). Cohesin's ATPase activity couples cohesin loading onto DNA with Smc3 acetylation. Curr. Biol. *24*, 2228–2237.

Michaelis, C., Ciosk, R., and Nasmyth, K. (1997). Cohesins: chromosomal proteins that prevent premature separation of sister chromatids. Cell *91*, 35–45.

Murayama, Y., and Uhlmann, F. (2015). DNA entry into and exit out of the cohesin ring by an interlocking gate mechanism. Cell *163*, 1628–1640.

Nasmyth, K., and Haering, C.H. (2005). The structure and function of SMC and kleisin complexes. Annu. Rev. Biochem. *74*, 595–648.

Nasmyth, K., and Haering, C.H. (2009). Cohesin: its roles and mechanisms. Annu. Rev. Genet. *43*, 525–558.

Nishiyama, T., Ladurner, R., Schmitz, J., Kreidl, E., Schleiffer, A., Bhaskara, V., Bando, M., Shirahige, K., Hyman, A.A., Mechtler, K., and Peters, J.M. (2010). Sororin mediates sister chromatid cohesion by antagonizing Wapl. Cell *143*, 737–749.

Rankin, S., Ayad, N.G., and Kirschner, M.W. (2005). Sororin, a substrate of the anaphase-promoting complex, is required for sister chromatid cohesion in vertebrates. Mol. Cell *18*, 185–200.

Rao, H., Uhlmann, F., Nasmyth, K., and Varshavsky, A. (2001). Degradation of a cohesin subunit by the N-end rule pathway is essential for chromosome stability. Nature *410*, 955–959.

Roig, M.B., Löwe, J., Chan, K.L., Beckouët, F., Metson, J., and Nasmyth, K. (2014). Structure and function of cohesin's Scc3/SA regulatory subunit. FEBS Lett. *588*, 3692–3702.

Rolef Ben-Shahar, T., Heeger, S., Lehane, C., East, P., Flynn, H., Skehel, M., and Uhlmann, F. (2008). Eco1-dependent cohesin acetylation during establishment of sister chromatid cohesion. Science *321*, 563–566.

Rowland, B.D., Roig, M.B., Nishino, T., Kurze, A., Uluocak, P., Mishra, A., Beckouët, F., Underwood, P., Metson, J., Imre, R., et al. (2009). Building sister chromatid cohesion: smc3 acetylation counteracts an antiestablishment activity. Mol. Cell *33*, 763–774.

Tanaka, K., Hao, Z., Kai, M., and Okayama, H. (2001). Establishment and maintenance of sister chromatid cohesion in fission yeast by a unique mechanism. EMBO J. *20*, 5779–5790.

Uhlmann, F., Lottspeich, F., and Nasmyth, K. (1999). Sister-chromatid separation at anaphase onset is promoted by cleavage of the cohesin subunit Scc1. Nature *400*, 37–42.

Uhlmann, F., Wernic, D., Poupart, M.A., Koonin, E.V., and Nasmyth, K. (2000). Cleavage of cohesin by the CD clan protease separin triggers anaphase in yeast. Cell *103*, 375–386.

Wilhelm, L., Bürmann, F., Minnen, A., Shin, H.C., Toseland, C.P., Oh, B.H., and Gruber, S. (2015). SMC condensin entraps chromosomal DNA by an ATP hydrolysis dependent loading mechanism in Bacillus subtilis. eLife *4*, http://dx.doi.org/10.7554/eLife.06659.

Molecular Cell, Volume *61*

Supplemental Information

Releasing Activity Disengages Cohesin's Smc3/Scc1 Interface in a Process Blocked by Acetylation

Frederic Beckouët, Madhusudhan Srinivasan, Maurici Brunet Roig, Kok-Lung Chan, Johanna C. Scheinost, Paul Batty, Bin Hu, Naomi Petela, Thomas Gligoris, Alexandra C. Smith, Lana Strmecki, Benjamin D. Rowland, and Kim Nasmyth

Figure S1

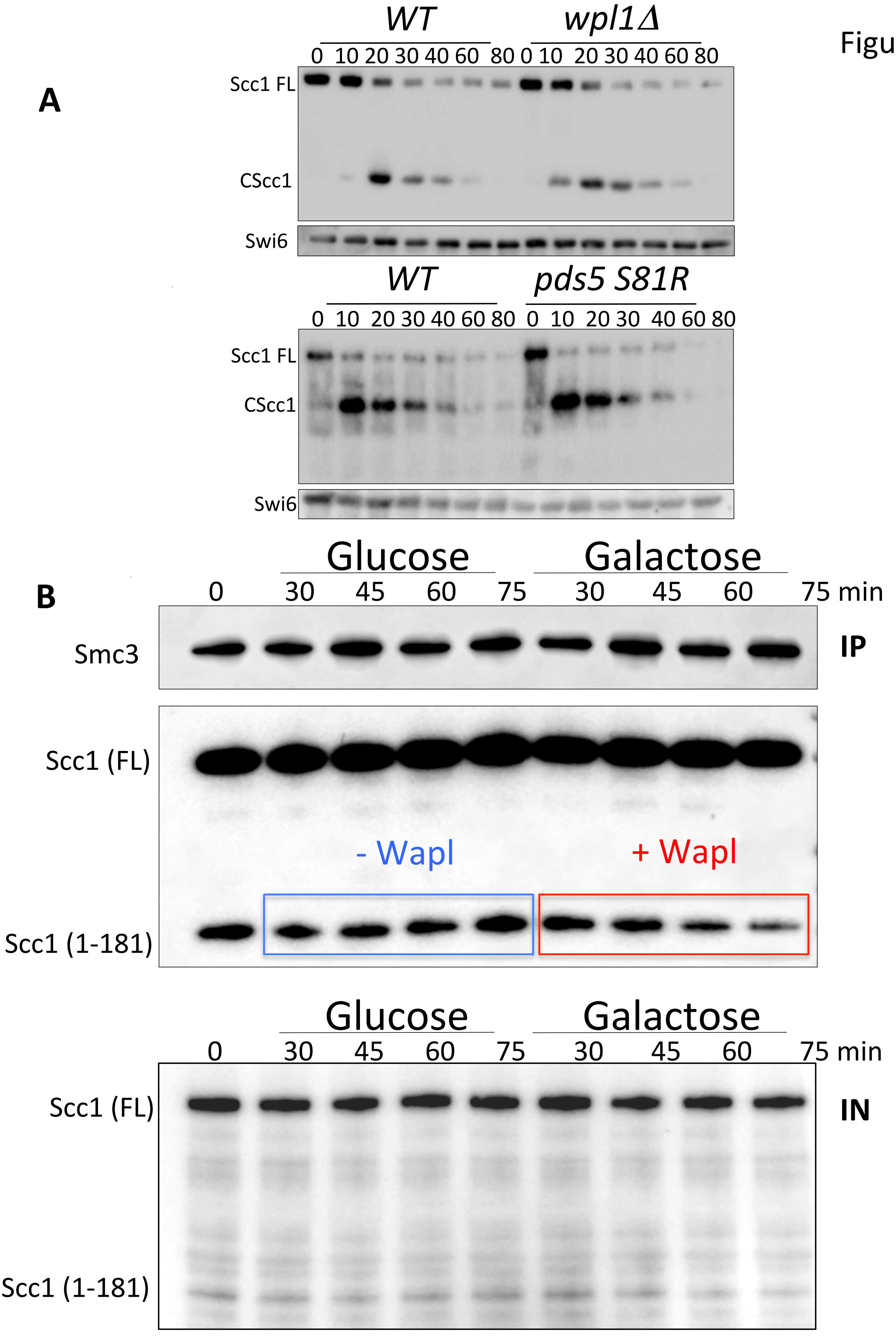
A
WT
wpl1Δ
0 10 20 30 40 60 80 0 10 20 30 40 60 80
Scc1 FL
CScc1
Swi6
WT
pds5 S81R
0 10 20 30 40 60 80 0 10 20 30 40 60 80
Scc1 FL
CScc1
Swi6
B
Glucose
Galactose
0 30 45 60 75 30 45 60 75 min
Smc3
IP
Scc1 (FL)
- Wapl
+ Wapl
Scc1 (1-181)
Glucose
Galactose
0 30 45 60 75 30 45 60 75 min
Scc1 (FL)
IN
Scc1 (1-181)

Figure S2

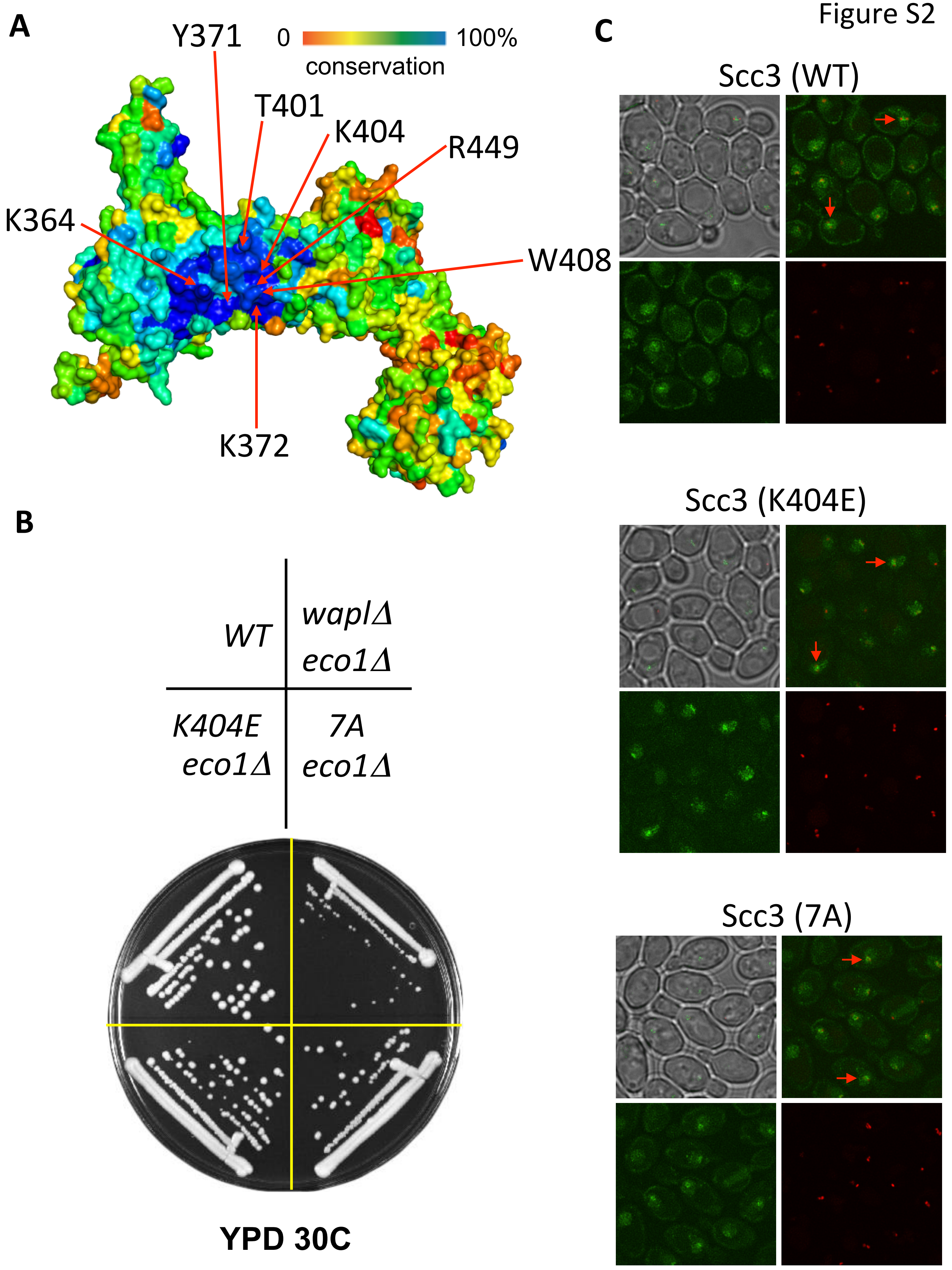
A
Y371
0
100%
conservation
T401
K404
R449
K364
W408
K372
B
WT
waplΔ
eco1Δ
K404E
eco1Δ
7A
eco1Δ
YPD 30C
C
Scc3 (WT)
Scc3 (K404E)
Scc3 (7A)

Figure S3

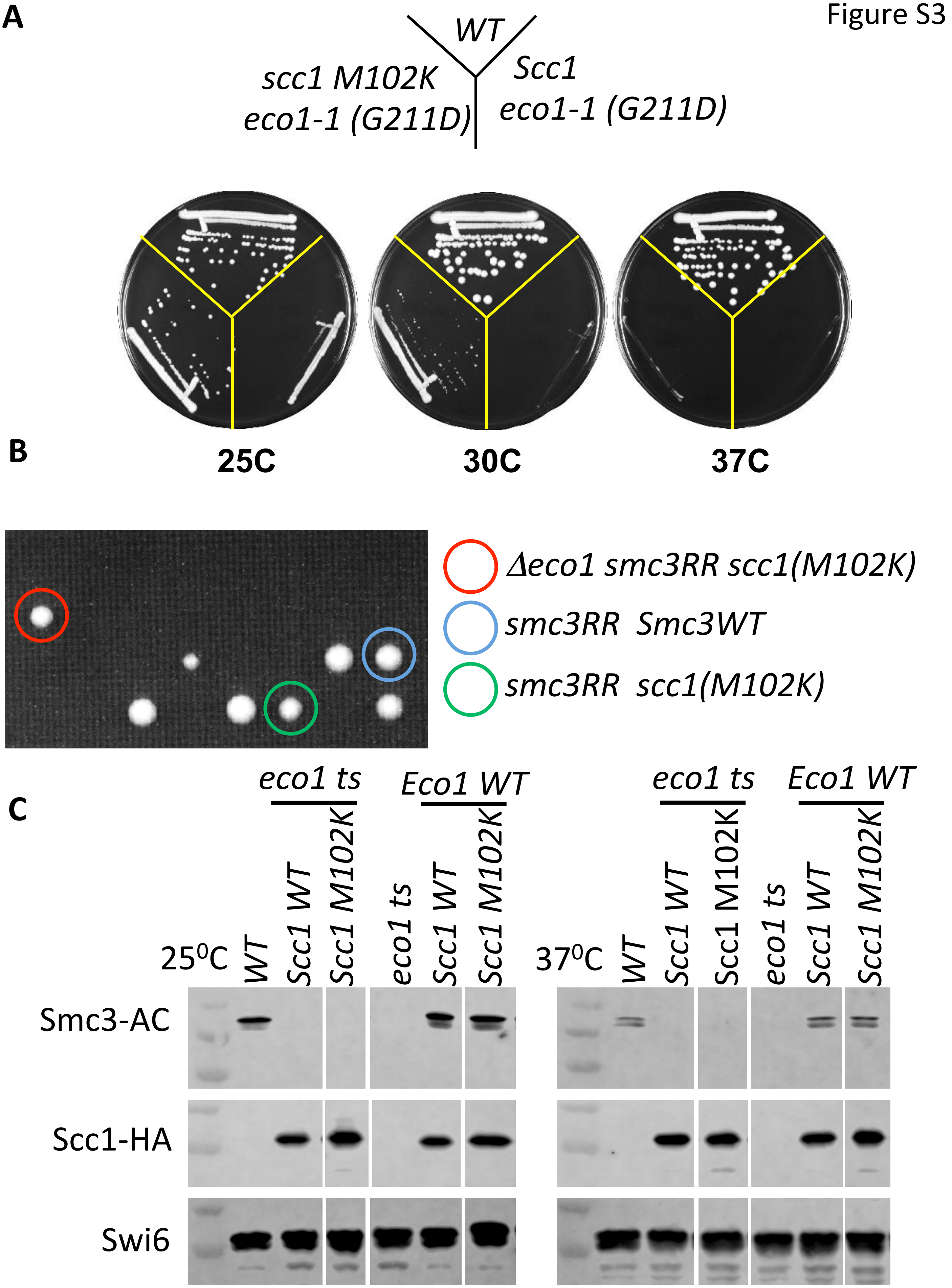
A
WT
scc1 M102K
eco1-1 (G211D)
Scc1
eco1-1 (G211D)
25C
30C
37C
B
Δeco1 smc3RR scc1(M102K)
smc3RR Smc3WT
smc3RR scc1(M102K)
C
eco1 ts
Eco1 WT
25⁰C
WT
Scc1 WT
Scc1 M102K
eco1 ts
Scc1 WT
Scc1 M102K
37⁰C
WT
Scc1 WT
Scc1 M102K
eco1 ts
Scc1 WT
Scc1 M102K
Smc3-AC
Scc1-HA
Swi6

Figure S4

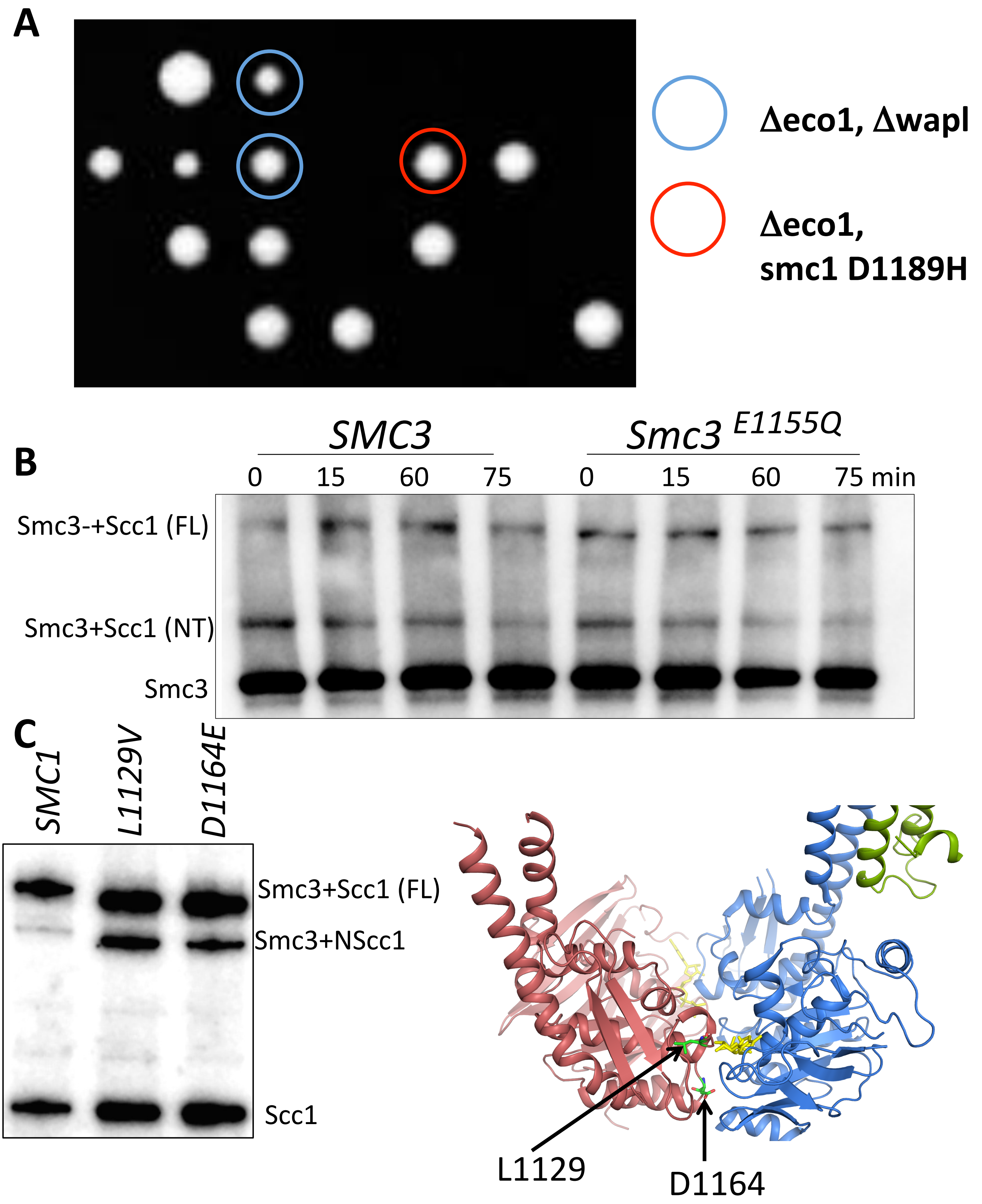
A
Δeco1, Δwapl
Δeco1,
smc1 D1189H
B
SMC3
Smc3 E1155Q
0
15
60
75
0
15
60
75 min
Smc3-+Scc1 (FL)
Smc3+Scc1 (NT)
Smc3
C
SMC1
L1129V
D1164E
Smc3+Scc1 (FL)
Smc3+NScc1
Scc1
L1129
D1164

Supplementary Figure Legends

Figure S1. Related to Figures 1 and 2

(A) Stability of Scc1 cleavage fragments in releasing activity mutants. Wild type (K17960), *wpl1Δ* (K20236) and *pds5-S81R* (K20521) strains expressing *CDC20* from the *GAL* promoter were grown to logarithmic phase at 25^0C in YP medium containing galactose , transferred to galactose free media to induce metaphase arrest (time 0), and anaphase triggered by galactose re-addition. Separase cleavage of Scc1 was followed by Western blotting, C-terminal HA epitopes.

(B) Wapl triggers dissociation of NScc1 from Smc3 HOS1 or *hos1Δ* strains containing galactose inducible *WPL1* gene, K22555 (*MATa SMC3 (S1043C)-HA6 MYC3-SCC1(TEV268) pGAL1-10-WPL1*) and K22810 (MAT*a hos1Δ SMC3(S1043C)-HA6 MYC3-SCC1(TEV268) pGAL1-10-WPL1*) were grown in YP Raffinose medium at 25^0C and were arrested in nocodazole for 2 hours. Galactose was then added to induce Wapl expression. Samples were taken at the indicated time points to immunoprecipitate Smc3 and analysed by Western blotting using anti MYC antibodies. The structure of Smc3-Scc1NTD complex PDB:4UX3 is shown with Scc1 C56, Smc3 S1043 residues marked.

Figure S2. Related to Figure 4. Scc3's highly conserved surface is essential for release

(A) Surface conservation of Scc3 orthologs projected on the surface of Zr *Scc3* (blue most and red least conserved) highlighting the conserved K364, Y371, K372, T401, K404, W408 and R449 (*S. cerevisiae*) surface residues that were mutated (7A). **(B)** A YPD plate with Strain K699 (W3031a), K16432 (*eco1Δ wpl1Δ*), K23905 (*eco1Δ scc3 K404E*) and K23908 (*eco1Δ scc3 7A*) grown at 30^0C shows the suppression of *eco1Δ* by *scc3* (*K404E* and *7A*) mutants. **(C)** Shows live cell imaging of strains K23851 (*WPL1-eGFP MTW1-RFP*), K23854 (*WPL1-eGFP MTW1-RFP scc3 K404E*) and K23852 (*WPL1-eGFP MTW1-RFP scc3 7A*) that were grown to exponential phase in YPD at 25^0C . GFP (Wapl) RFP (Mtw1) and DIC channels are shown.

Figure S3. Related Figure 5. Role of residues within Scc1's NTD in the release mechanism

(A) YPD plates with Strain K699 (W3031a), K24507 (*SCC1-HA eco1−1(G211D)*), and K24514 (*SCC1 M102K-HA eco1−1(G211D)*) were grown at 25^{0}C, 30^{0}C and 37^{0}C. **(B)** Diploid (*MATa/α SMC3 smc3Δ TRP1::smc3 K112R K113R Eco1 eco1Δ scc1Δ SCC1 M102K*) was sporulated, tetrads dissected, and selected haploid segregants with their genotypes shown. **(C)** Exponentially growing strains K699 (W3031a), K20307 (*SCC1-HA eco1-1*), K20311 (*SCC1M102K-HA eco1-1*), K16300 (*eco1-1*), K18879 (*SCC1-HA*) and K18881 (*SCC1M102K-HA*), grown at either permissive 25^{0}C or the non permissive 37^{0}C were lysed and the levels of Smc3 acetylation (Smc3-AC) was detected using antibody that recognises Smc3 K112 K113 AC. Scc1 levels was measured using anti HA antibody and Swi6 was used as loading control. The data are from the same western blot with irrelevant lanes removed.

Figure S4. Related to Figure 5 and 7

(A) Role of residues within Smc3's ATPase domain in release

Diploids (*MATa/α scc1Δ LEU2::MYC-SCC1 TRP1::smc3 K112R K113R D1189H eco1Δ wpl1Δ*) was sporulated, tetrads dissected, and selected haploid segregants with their genotypes shown.

(B) Disengagement of NScc1 from Smc3 requires a single round of ATP hydrolysis

Strains K23567 (*MATa SMC3 URA3::SMC3 S1043C-HA6 MYC3-SCC1(TEV268) pGAL1-10-WPL1*) and K23566 (*MATa SMC3 URA3::smc3(S1043C E1155Q-HA6 MYC3-SCC1(TEV268), pGAL1-10-WAPL*) were grown in YP Raffinose medium at 25^{0}C and were arrested in nocodazole for 2 hours. Galactose was then added to induce Wapl expression. Samples were taken at the indicated time points to induce *in vivo* cross-linking with 5mM BMOE. Cross-linking was analysed by Western blotting using anti HA antibodies.

(C) Disengagement of NScc1 from Smc3 involves engagement of Smc ATPase heads

Exponentially growing strains K24297 (*MATa SMC3 S1043C::ADE2 MYC3-SCC1(TEV268) SMC1)*, K24523 (*MATa SMC3 S1043C::ADE2 MYC3-SCC1(TEV268) smc1 L1129V)* and K24521 (*MATa SMC3 S1043C::ADE2 MYC3-*

SCC1(TEV268) smc1 D1164E) were treated as described in Figure 6(C) and analysed by western blotting using anti MYC antibodies. The positions of the Smc1 L1129 and D1164 residues are marked in a model of Smc1 and Smc3 head domains that are in an engaged state.

Strain construction

The 7A mutant version of scc3 (under its native promoter) was incorporated at the leu2 locus in heterozygous SCC3/scc3Δ diploids cells. Diploids were sporulated and tetrads dissected at 23 °C on YPD plates. The genotype of the resulting haploids was determined by replica plating, and viable cells with only the ectopic copy were used in this manuscript. The single mutants K404E, K404A, Y405E and Y405A on scc3 were generated modifying the genome in *S.cerevisiae* using the CRISPR-Cas9 systems. For this we co-transformed the cells with a 600nt PCR "healing fragment" along with a 2 micron LEU2 plasmid constitutively expressing Cas9 endonuclease and transcribing the structural RNA that contains the Cas9 binding site and the guide RNA that will target the double strand break formation by Cas9 to the scc3 gene. The guide RNA was designed 20nt upstream of the NGG (L455, E456) using the annealed oligos MB530F/MB530R (MB530F: 5'-ATCACGCTTCAAAACTAAGATCC-3' , MB531R: 5'- AACGGATCTTAGTTTTGAAGCGT-3') and fused afterwards at the 5' end of the structural RNA with a Sap1 cloning. The 600nt PCR "healing fragment" containing the single mutants, a mutagenized NGG Cas9 sequence target (L455, E456: C**TGG**AA to C**TAG**AA) and 5'-, 3'- homology arms by which to repair the double strand break created by Cas9 was created using the oligos MB528F/MB529R (MB528F: 5-CTTGTGTTGGATTTGTTGAC-3', MB529R: 5'-TGGATACTGTGGATCCTTACATCAAGATTAACGTCACGGATTGCCACTTCT AGGATCTTAGTTTTGAAGCG-3'). As a control, no colonies were obtained after yeast transformation of the 2 micron Leu2 plasmid alone without the "healing fragment". All mutations were confirmed afterwards by genome sequencing.

In vivo chemical cross-linking

Strains were grown in YEPD at 25 °C to OD_{600nm} = 0.5-0.6 and arrested in nocodazole for 1.5 h. 30 OD units were washed in ice-cold PBS, then re-suspended in 1.5 ml ice-cold PBS. The suspensions were then split into 2 x 750 μl and 35 μl BMOE (stock: 125 mM in DMSO, 5 mM final) or DMSO was added for 6 min on ice. Cells were washed with 2 x 2 ml ice-cold PBS containing 5 mM DTT, resuspended in 500 μl EBX lysis buffer containing 1 mM DTT, benzonase (1:1000), RNase (0.1 mg/ml), Roche Complete Protease Inhibitors (2X) and PMSF (1 mM), lysed in a FastPrep-24 (MP Biomedicals) for 3 min at 6 m/s with 500 μl of acid-washed glass beads (425-

600 μm, Sigma) and lysates cleared (5 min, 12 k*g*). Protein concentrations were adjusted after Bradford assay and cohesin immuno-precipitated using anti-PK antibody (AbD Serotec, 1 h, 4 °C), anti MYC antibody (Santa cruz1 h, 4 °C), and protein G dynabeads (1 h, 4 °C with rotation) or Anti HA affinity resin (Roche, 1 h, 4 °C). Beads were washed with 2 x 1 ml lysis buffer, resuspended in 50 μl 2x sample buffer, incubated at 65°C for 5 min and the supernatant loaded onto a either 3-8% or 4-12% gradient gels (life technologies).

Mini Chromosome IPs

Strains containing a 2.3KB minichromosome were grown in YEPD at 25 °C and processed for in vivo crosslinking like mentioned above. For Southern blotting, pull-downs of lysates (in 25 mM Hepes pH 8.0, 50 mM KCl, 50 mM $MgSO_4$, 10 mM trisodium citrate, 25 mM sodium sulfite, 0.25% triton-X, Roche Protease Inhibitor, 1 mM PMSF, 2 mM DTT, 0.1 mg/ml RNase) containing a circular 2.3 kb mini-chromosome were performed as above, DNA was eluted in 30 μl 1% SDS with DNA loading dye (65 °C, 4 min) and run on 0.8% agarose gels containing ethidium bromide (1.4 V/cm, 22h, 4 °C).

Protein Purification

E. coli BL21(DE3) RIPL cells (Stratagene) were transformed with pET vectors containing the *S. cerevisiae* Scc3 WT, Scc3 E404K and Wapl genes and induced at OD_{600} 0.6 with 1 mM IPTG at 23 °C over night. The cells were pelleted and re-suspended with TAP buffer (50 mM Tris–HCl, 250 mM NaCl, 1 mM β-mercaptoethanol, pH 7.5) adding EDTA-free protease inhibitor cocktail tablets (Roche). After re-suspension, and one round of cell lysis at 18 KPsi (Constant Systems), 1 min sonication at 80% AMPL (Sonics Vibra-Cell) were performed. Cell lysate was pre-cleared by centrifugation at 4000 rpm for 15 min, and supernatant was cleared by centrifugation at 15,000 rpm for 1 h at 4 °C (Beckman Coulter, rotor JLA-16.250). All purified proteins are His_6 tagged and supernatants were incubated with Talon Superflow beads (Clontech) for 3 h at 4 °C. Beads were washed 3 times with TAP buffer containing 10 mM imidazole. Proteins were eluted in TAP buffer with 500 mM imidazole and loaded onto a Superdex 200 16/60 chromatography column

(GE Healthcare) equilibrated with TAP buffer (50 mM Tris–HCl, 100 mM NaCl, 1 mM β-mercaptoethanol, pH 7.5). Peak fractions were collected and concentrated using Vivaspin columns (Sartorius Stedim Biotech).

Gel filtration for Protein Binding

For in vitro binding assays, 15 μM of each pure protein were incubated at 4 °C for 2–3 h, at a final volume of 150 μl in TAP buffer (50 mM Tris–HCl, 100 mM NaCl, 1 mM β-mercaptoethanol, pH 7.5) and then loaded onto a Superose 6 (GE Healthcare) column. 15 μl protein fractions were collected and analyzed by 3-8% SDS–PAGE and Coomassie staining.
